# The Effect of Sedentary Behaviour on Cardiorespiratory Fitness: A Systematic Review and Meta-Analysis

**DOI:** 10.1007/s40279-023-01986-y

**Published:** 2024-01-16

**Authors:** Stephanie A. Prince, Paddy C. Dempsey, Jennifer L. Reed, Lukas Rubin, Travis J. Saunders, Josephine Ta, Grant R. Tomkinson, Katherine Merucci, Justin J. Lang

**Affiliations:** 1 Centre for Surveillance and Applied Research, Public Health Agency of Canada, 785 Carling Avenue, Ottawa, ON K1A 0K9 Canada; 2 School of Epidemiology and Public Health, Faculty of Medicine, University of Ottawa, Ottawa, ON Canada; 3 Institute for Physical Activity and Nutrition (IPAN), School of Exercise and Nutrition Sciences, Deakin University, Geelong, VIC Australia; 4 MRC Epidemiology Unit, Institute of Metabolic Science, University of Cambridge, Cambridge Biomedical Campus, Cambridge, UK; 5 Diabetes Research Centre, College of Life Sciences, University of Leicester, Leicester, UK; 6 Baker Heart and Diabetes Institute, Melbourne, VIC Australia; 7 Exercise Physiology and Cardiovascular Health Lab, University of Ottawa Heart Institute, Ottawa, ON Canada; 8 School of Human Kinetics, Faculty of Health Sciences, University of Ottawa, Ottawa, ON Canada; 9 Department of Physical Education and Sport, Faculty of Science, Humanities and Education, Technical University of Liberec, Liberec, Czech Republic; 10 Institute of Active Lifestyle, Faculty of Physical Culture, Palacký University Olomouc, Olomouc, Czech Republic; 11 Department Applied Human Sciences, University of Prince Edward Island, Charlottetown, PEI Canada; 12 Telfer School of Management, University of Ottawa, Ottawa, ON Canada; 13 Alliance for Research in Exercise, Nutrition and Activity, Allied Health and Human Performance, University of South Australia, Adelaide, SA Australia; 14 Health Canada Library, Ottawa, ON Canada

## Abstract

**Background:**

Cardiorespiratory fitness (CRF) is an important indicator of current and future health. While the impact of habitual physical activity on CRF is well established, the role of sedentary behaviour (SB) remains less understood.

**Objective:**

We aimed to determine the effect of SB on CRF.

**Methods:**

Searches were conducted in MEDLINE, Embase, PsycINFO, CINAHL and SPORTDiscus from inception to August 2022. Randomised controlled trials, quasi-experimental studies and cohort studies that assessed the relationship between SB and CRF were eligible. Narrative syntheses and meta-analyses summarised the evidence, and Grading of Recommendations, Assessment, Development and Evaluation (GRADE) certainty was based on evidence from randomised controlled trials.

**Results:**

This review included 18 studies that focused on youth (four randomised controlled trials, three quasi-experimental studies, 11 cohort studies) and 24 on adult populations (15 randomised controlled trials, five quasi-experimental studies, four cohort studies). In youth and adults, evidence from randomised controlled trials suggests mixed effects of SB on CRF, but with the potential for interventions to improve CRF. Quasi-experimental and cohort studies also support similar conclusions. Certainty of evidence was very low for both age groups. A meta-analysis of adult randomised controlled trials found that interventions targeting reducing SB, or increasing physical activity and reducing SB, had a significant effect on post-peak oxygen consumption (mean difference = 3.16 mL^.^kg^–1.^min^–1^, 95% confidence interval: 1.76, 4.57).

**Conclusions:**

Evidence from randomised controlled trials indicates mixed associations between SB and CRF, with the potential for SB to influence CRF, as supported by meta-analytical findings. Further well-designed trials are warranted to confirm the relationship between SB and CRF, explore the effects of SB independent from higher intensity activity, and investigate the existence of such relationships in paediatric populations.

**Clinical Trial Registration:**

PROSPERO CRD42022356218.

**Supplementary Information:**

The online version contains supplementary material available at 10.1007/s40279-023-01986-y.

## Key Points

**Table:** 

Evidence from randomised controlled trials indicates mixed associations between sedentary behaviour and cardiorespiratory fitness, with the potential for reduced sedentary behaviour to increase cardiorespiratory fitness, as supported by meta-analytical findings.
Further well-designed trials are warranted to confirm the relationship between sedentary behaviour and cardiorespiratory fitness, explore the effects of sedentary behaviour independent from higher intensity activity and to investigate the existence of such relationships in pediatric populations.

## Introduction

Cardiorespiratory fitness (CRF) is an important measure of health-related fitness and refers to the capability of the cardiovascular and respiratory systems to supply oxygen to muscles for energy production during continuous, large-muscle, whole-body physical activity [1]. Cardiorespiratory fitness is a good indicator of habitual aerobic activity [2–4], and engaging in a greater volume of aerobic activity beyond activities of daily living (e.g. structured exercise and physical training, sports) shown to improve CRF [5, 6]. Importantly, while all physical activity appears to positively influence CRF, some evidence suggests that activity intensity is an important factor [7] with vigorous-intensity physical activity more strongly associated with CRF than light-intensity and moderate-intensity physical activity [1, 8–13].

Cardiorespiratory fitness is a useful prognostic indicator for the health of a population [14, 15] and is predictive of future health outcomes, including cardiometabolic health [16, 17], cardiovascular disease [18, 19], cardiovascular mortality [20, 21], cancer mortality [19, 22] and all-cause mortality [18, 19, 23–26]. Cardiorespiratory fitness is linked to mortality independently of a number of important covariates including age, sex, smoking, alcohol consumption, self-reported physical activity levels, socioeconomic status and comorbidities, making it a powerful predictor of future health states and death [18, 19, 24, 27, 28]. In fact, no other modifiable risk factor has been shown to be a stronger independent predictor of health (e.g. cardiometabolic disease) and longevity than CRF [1, 20, 28, 29].

While habitual physical activity, specifically vigorous intensity physical activity, has long been established as an important contributor to CRF [1, 8], much less is known about the other end of the activity continuum, including sedentary behaviour (SB). Sedentary behaviour is not synonymous with physical inactivity (i.e. insufficient moderate-to-vigorous intensity physical activity [MVPA]), but a distinct behaviour defined by activities undertaken at a low energy expenditure (≤ 1.5 metabolic equivalents of task [METs]) while sitting, lying or reclining [30]. Levels of SB, especially sedentary screen time, have continued to rise [31–34], while CRF has been declining over time [35]. Given that many countries observed increases in population levels of SB during the coronavirus disease 2019 pandemic [36–38] and declines in CRF [39, 40], it is essential to understand the direct relationship between SB and CRF.

Cross-sectional evidence suggests that higher volumes of SB are associated with lower CRF in youth [41–44] and adults [45–49]. A recent systematic review of cross-sectional studies of adults found that device-assessed sedentary time was negatively, though weakly, associated with CRF (r =  − 0.16, 95% confidence interval [CI]: − 0.24, − 0.09) [48]. Recently, there has been an increase in the number of randomised controlled trials (RCTs) assessing the efficacy of SB interventions to increase CRF either as a primary or secondary outcome [50–53]. No such review has focused on RCTs (to establish causality) or considered the effects of SB independent of physical activity. Therefore, the primary objective of this systematic review was to determine the effect of SB on CRF. A secondary objective was to understand whether the effect of SB on CRF is independent of MVPA.

## Methods

A systematic review was used to identify all studies that reported on the effects of SB (e.g. sedentary time, sitting time, screen time) on CRF outcomes. To meet the objective of this review, RCTs were the primary study design considered, but quasi-experimental and cohort studies were also explored to supplement RCT findings. The review adheres to the Preferred Reporting Items for Systematic Reviews and Meta-Analyses (PRISMA) statement [54] and was prospectively registered on PROSPERO (CRD42022356218).

### Inclusion Criteria

#### Population

All in-vivo human studies were eligible. Pregnant populations were ineligible.

#### Exposures

Sedentary behaviour includes activities undertaken at a low energy expenditure (≤ 1.5 METs) while sitting, lying or reclining [30]. Intervention studies were required to include an intervention component that targeted total or type-specific SB or the disruption (break-up) of prolonged SB. Co-interventions (e.g. including a physical activity, diet, weight loss component) were eligible, but were required to provide a quantification of time spent sedentary. A minimum follow-up time of ≥ 7 days was required to exclude studies that assessed immediate/acute effects on CRF. Cohort studies were required to assess exposure to SB at baseline or changes in SB over time and their subsequent effects on CRF.

Sedentary behaviour could be measured either via self-report (e.g. questionnaire, diary/log) or by device (e.g. accelerometer, inclinometer) and could include total time spent sedentary, sitting or in a specific sedentary activity (e.g. screen time, watching television, reading, using a computer or electronic device, playing video games).

#### Controls

Randomised controlled trials were required to have a no-intervention comparison arm (e.g. usual care, attention control, no intervention). Other studies included historical or comparator controls (e.g. pre-post single-arm trial, multi-arm trial without controls, prospective cohorts, case–control) to establish some element of causality.

#### Outcomes

Cardiorespiratory fitness could be measured directly or indirectly. Direct measures of CRF include maximal or peak oxygen consumption during exercise testing (hereafter called *V̇O*_*2*_*peak*). Indirect estimates included prediction equations with a variety of inputs, including age, sex, body mass index, height and aerobic exercise performance (i.e. test time, distance, physiological response). Additionally, indirect measures included resting heart rate, maximal heart rate, exercise performance/tolerance (e.g. distance covered, laps, running speed, test duration) and efficiency/economy (heart rate response to exercise, METs). Cardiorespiratory fitness testing could be maximal or submaximal using a variety of modalities such as cycling, running, walking or bench stepping.

#### Study Designs

Randomised controlled trials were required to have a no-intervention comparison arm. Other study designs included those with historical or comparator controls such as retrospective cohort studies, prospective cohort studies, pre-post studies (quasi-experimental studies) and case–control studies. These study designs provide sufficient detail to examine causal associations for changes in SB and its impact on CRF.

#### Study Language, Publication Status and Timeframe

No language restrictions were included in the search strategy, but only publications in English, French, Spanish and Czech were included based on authors’ language capacity. Only published peer-reviewed studies were eligible. All literature regardless of date of publication was considered.

### Search Strategy

A comprehensive search strategy was developed in collaboration with a research librarian (KM) from the Health Canada Library with input from review authors. The primary search was created in MEDLINE and tested to ensure the capture of previously identified key papers. An RCT filter was used and adapted from the *Cochrane Handbook for Systematic Reviews of Interventions* [55]. The MEDLINE search was peer reviewed by a Health Canada librarian using the PRESS peer review guidelines [56]. KM completed the searches in MEDLINE® All (via Ovid), Embase (via Ovid) and Scopus. A research librarian from the University of Ottawa (VL) translated and completed the searches in CINAHL (via EBSCOhost) and SPORTDiscus (via EBSCOhost). All searches were run from database inception. The CINAHL and SPORTDiscus searches for non-RCTs were exported on 31 August, 2022. All remaining searches were exported on 29 August, 2022 (Table S1 of the Electronic Supplementary Material [ESM]). Bibliographies of topical systematic reviews evaluating SB and health outcomes that included CRF were scanned for additional studies.

### Article Screening

Articles were imported into EndNote, where duplicates were removed. Screening was conducted in Covidence, which also automatically identified and removed duplicates. Two independent reviewers (two of the following authors: SAP, PCD, JLR, LR, TJS, JT, GRT or JJL) screened the titles and abstracts of all studies to identify potentially relevant articles. The full texts of all studies that either met the inclusion criteria or provided insufficient information in the abstract to exclude were obtained and reviewed. Two independent reviewers (two of the following authors: SAP, PCD, JLR, LR, TJS, JT, GRT or JJL) screened the full texts for inclusion. If conflicts arose, discussion between the reviewers and a possible third reviewer was conducted to achieve a final decision.

Two rounds of article screening were conducted. First, the search strategy used a filter to only include RCTs because this was considered the gold standard of evidence for the research question. Limited RCT evidence was identified resulting in a second expanded search that included articles originally excluded by the RCT filter. This resulted in a second round of screening that captured quasi-experimental studies and cohort studies.

### Data Extraction and Analysis

Data extraction forms were completed in Covidence by two independent reviewers (SAP, PCD, JLR, LR, TJS, JT, GRT or JJL) with conflicts resolved by a third (SAP or JJL). The reviewers were not blinded to the authors or journals when screening or extracting data but did not extract data from their own work.

A narrative synthesis, including summary tables, was used to summarise findings across all studies and grouped by outcome. When enough RCTs were available (more than two studies), pooled effects of SB interventions on each outcome were estimated using a random-effects (Der Simonian Laird method) meta-analysis for mean differences (using post-values or changes, depending on reporting of data in primary studies). When more than one intervention was compared to a single control group, the control group sample was divided by the number of intervention groups. Heterogeneity was assessed using the chi-squared test and I^2^ statistic. Publication bias was not assessed as fewer than ten studies were included in each meta-analysis [57, 58]. Leave-one-out sensitivity analyses were conducted to determine if removing an individual study had a meaningful impact on the final effect estimates. Forest plots and meta-analyses were created using Review Manager (RevMan) 5.4 (The Cochrane Collaboration, 2020).

To assess the secondary objective of SB on CRF independent of MVPA, we conducted a subgroup analysis comparing RCTs that did and did not include a physical activity focus/component. Additionally, subgroup analyses explored the population group (i.e. apparently healthy vs condition/disease population), CRF outcome measurement method (i.e. direct vs indirect method), average CRF levels at baseline (age-specific and sex-specific low CRF: ≤ 50th percentile vs high CRF > 50th percentile [59]; female percentiles were used if the sample included both male and female individuals), degree of intervention effectiveness for sedentary behaviour (e.g. intervention had a statistically significant effect on overall sedentary time in hour/day), intervention duration (e.g. ≤ 3 months/3–6 months/ > 6 months) and study risk of bias scores (i.e. low, some, high). Non-RCT interventions were not included in the meta-analysis because of high levels of heterogeneity in the methodology and reporting of results.

### Risk of Bias Appraisal for Individual Studies

The risk of bias of the individual studies was assessed based on study design. Version 2 of the Cochrane Collaboration’s Tool for Assessing Risk of Bias (RoB 2) was used for randomised trials [60, 61], the Risk of Bias in Non-randomized Intervention Studies (ROBINS-I) tool for quasi-experimental studies [62] and the Risk Of Bias in Non-randomized Studies—of Exposure (ROBINS-E) tool for observational studies [63]. Risk of bias assessments were carried out by two independent reviewers (SAP, PCD, JLR, LR, TJS, JT, GRT or JJL). Conflicts were resolved by a third reviewer (SAP or JJL).

### Grading of the Overall Evidence

The certainty and strength of the evidence were assessed using a modified Grading of Recommendations, Assessment, Development and Evaluation (GRADE) approach [64]. The certainty of the evidence was categorised as high, moderate, low or very low. Randomised controlled trials provided evidence starting at high certainty, while observational studies began at low certainty. For this systematic review, the GRADE assessment was based solely on the evidence from RCTs. Certainty was determined based on confidence in the effect estimate and adjusted considering limitations in study design or execution, inconsistency of results, indirectness of evidence and imprecision (see Table S2 of the ESM for a summary of decision rules). One reviewer (SAP) assessed the evidence for each outcome, and a second reviewer (JJL) verified the assessment for accuracy. Disagreements were resolved through group discussion and consensus. Evidence profiles are presented using summary of findings tables.

## Results

### Study Characteristics

**Fig. 1:**
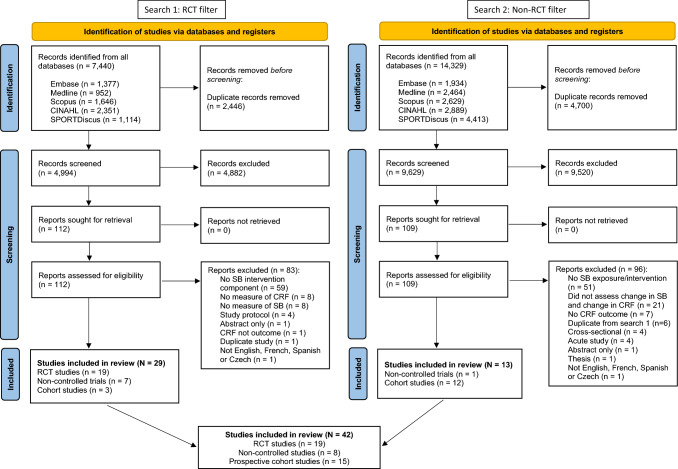
Preferred Reporting Items for Systematic Reviews and Meta-Analyses (PRISMA) flow diagram for the identification, screening and inclusion of studies. *CRF* cardiorespiratory fitness, *SB* sedentary behaviour, *RCT* randomised controlled trial

Figure 1 provides a detailed flow diagram of the literature search and screening process including reasons for a full-text exclusion. The two search rounds identified 21,769 potentially relevant papers. Of these, 3416 were identified in MEDLINE, 3311 in Embase, 4275 in Scopus, 5240 in CINAHL and 5527 in SPORTDiscus. After deduplication, a combined 14,623 relevant papers remained. Title and abstract review resulted in 221 full-text papers for assessment. Of these, 19 RCTs [50, 51, 65–81], eight quasi-experimental studies [82–89] and 15 cohort studies [11, 90–102] met the study inclusion criteria. A list of excluded full texts and reasons can be found in Table S3 of the ESM. Individual study characteristics can be seen in Tables S4–S6 of the ESM.

Eighteen studies focused on youth (four RCTs, three quasi-experimental studies, 11 cohort studies) and 24 on adults (15 RCTs, five quasi-experimental studies, four cohort studies). Included studies were published between 1999 and 2022; the majority (67%) in the past 5 years. The evidence was obtained from 17 countries/regions, with the USA (28%) and Australia (12%) being the most frequent.

Nineteen RCTs examined the influence of a SB intervention on CRF [50, 51, 65, 67–81, 103]. In youth, three RCTs took place in schools, and one was among children with obesity in a community setting. In adults, most (60%) of the RCT evidence included adults working in sedentary occupations in office settings. Seven had at least one intervention arm that only targeted SB (vs a combination of SB plus physical activity), 16 targeted the reduction in total or domain-specific SB (e.g. school, occupational) and most used an educational intervention. In youth, the intervention duration was similar with a range between 6 and 8 months. In adults, the RCT intervention length ranged from 4 weeks to 3 years, with most (60%) lasting 12 weeks or less. Most RCTs targeted non-clinical populations and included both sexes. The majority of RCTs included CRF as a secondary outcome. In youth, laps completed on the 20-m shuttle run test was the most common measure of CRF, while in adults the most common was V̇O_2_peak (mL^.^kg^−1.^min^–1^).

### Risk of Bias

Risk of bias for the individual studies is summarised in Tables S7a–c of the ESM. Fourteen of the RCTs (some concerns to high), all quasi-experimental studies (serious or critical), and seven of the cohort studies (high or very high) were identified as having a moderate to high risk of bias. Of the RCTs, the majority had some or a high risk of bias owing to deviations from the intended interventions (72%) and missing outcome data (56%). Among the quasi-experimental studies, most suffered from a serious or critical risk of bias due to confounding (75%) and the measurement of outcomes (50%). Of the cohort studies, the largest sources of bias included a lack of control for important confounding factors (40%) and missing data (47%).

### RCT Evidence

**Fig. 2:**
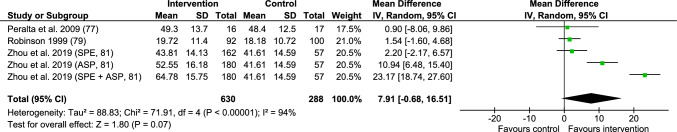
Meta-analysis comparing post-values of laps completed on the 20-m shuttle run in intervention groups with control groups in youth. *ASP* afterschool programme intervention, *CI* confidence interval, *SD* standard deviation, *SPE* school physical education intervention, *SPE* + *ASP* school physical education and afterschool programme intervention

Tables S8 and S9 of the ESM present the individual study findings for the RCTs. Tables 1 and 2 presents the summary of findings and certainty of the evidence for youth and adults, respectively. In youth, there was evidence of mixed effects of SB on CRF with two studies reporting no significant between-group difference [77, 79] and two reporting a significant improvement in CRF [80, 81] in the intervention compared with the control group. A meta-analysis of three RCTs (Fig. 2) found there was no statistically significant effect of SB interventions on post-values for laps completed on the 20-m shuttle run test (mean difference [MD] = 7.9 laps, 95% CI: − 0.7, 15.5, *p* = 0.07). The intervention effects reported by Zhou et al. [81] for the two arms that targeted physical activity in an afterschool programme (ASP and ASP plus school physical education) were significantly higher than the school physical education-targeted arm, and the other RCT evidence. Removal of these arms reduced the heterogeneity from 94 to 0%. The quality of evidence from RCTs assessing youth was downgraded to very low because of a risk of bias (one study had a high risk of bias and two with concerns), inconsistency (one trial [81] reported different effects) and indirectness (due to variations in populations and co-interventions).

**Table 1:** Summary of findings for SB and CRF in youth

Study design	Effect estimates or summary of effect^a^	# of participants (# of studies)	Certainty (quality) of evidence	Interpretation of findings
RCT	**CRF measured via laps completed on the 20-m shuttle run** [77, 79, 81] Pooled mean difference post-values for intervention vs. control: 7.91, 95% CI: − 0.65, 16.47, *p* = 0.07 All three studies targeted reduced screen time (two with a PA component [77, 81]). Two studies observed a significant decrease in screen time vs. control [79, 81], the other did not [77]. Only one [81] saw a significant change in PA. **CRF measured via recovery HR** [80] NS difference between groups (no difference in PA or SB)	**Laps** I: 618, C: 288 (3) **Recovery HR** I: 60, C: 56 (1)	**Very low certainty** RoB: − 2 points, one study had high RoB, 2 had some concerns Inconsistency: − 1 point, effects from one trial differed, but could likely be explained by intervention target Indirectness: − 2 points, variation in population and co-interventions Imprecision: 0 point, OIS met	There is very low certainty of mixed effects of SB on CRF. Note: 3/4 trials targeted PA, 3/4 trials significantly reduced SB
Quasi-experimental studies	**CRF measured via V̇O**_**2**_**peak** [83, 85] One study [83] targeted reduced total SB and found a significant increase in CRF (like the study arms that targeted PA). The other [85] targeted reduced leisure screen time and found that while SB decreased significantly, MVPA also increased significantly and change in CRF was largely correlated with change in MVPA. **CRF measured via resting and recovery HR** [86] Both Black and non-Black students saw improvements in their leisure screen time and PA levels. Resting HR only significantly reduced in non-Black students, while recovery HR significantly improved in Black students only.	**V̇O**_**2**_**peak** 120 (2) **Resting/recovery HR** 3813 (1)	NA	Evidence from quasi-experimental studies is mixed (1 positive, 1 mixed, 1 NS).
Cohorts	**CRF measured via V̇O**_**2**_**peak** [93, 94, 97, 99, 102] 3/5 studies (only 1 controlled for PA) observed that reduced screen time or total SB was not significantly associated with CRF [94, 99, 102]. Two studies (1 controlled for PA) found that lower screen time was associated with greater CRF [93, 97]. **CRF measured via laps completed on the 20-metre shuttle run** [90, 91, 100, 104, 105] 3/5 studies (only 1 controlled for PA) observed that reduced SB was significantly associated with greater CRF [90, 91, 104]. One study found no significant association [105]. One study found that greater SB was associated with greater CRF [100]. **CRF measured via running distance** [98] Evidence suggests a significant association between higher TV watching and reduced CRF.	**V̇O**_**2**_**peak** 4171 (5) **Laps** 4071 (5) **Distance** 135 (1)	NA	Evidence from cohort studies is mixed (6 positive, 4 NS, 1 negative). Three studies controlled for PA in the analysis, and the direction of association differed within each one.

*C* control group, *CI* confidence interval, *CRF* cardiorespiratory fitness, *HR* heart rate, *I* intervention group, *MET* metabolic equivalents of task, *MVPA* moderate-to-vigorous intensity physical activity, *NA* not applicable, *NS* non-significant, *OIS* optimal information size, *PA* physical activity, *RCT* randomised controlled trial, *RoB* risk of bias, *SB* sedentary behaviour, *TV* television

^a^See supplementary Tables 8, 10 and 12 for detailed results

**Table 2:** Summary of findings for SB and CRF in adults

Study design	Effect estimates or summary of effect	# of participants (# of studies)	Certainty (quality) of evidence	Interpretation of findings
RCT^a^	**CRF measured via V̇O**_**2**_**peak **[50, 51, 71, 73, 75, 76, 103] Pooled mean difference post-values for V̇O_2_peak intervention vs. control: 3.16 mL·kg^−1^·min^−1^, 95% CI: 1.76 to 4.57, *p* < 0.00001 SB-only: 2.18, 95% CI: 0.01 to 4.36, *p* = 0.05 SB + PA: 4.29, 95% CI: 2.87 to 5.70, *p* < 0.00001 **CRF measured via running distance **[70] NS effect of SB-only intervention on CRF **CRF measured via resting HR** [67–69, 72] Pooled mean difference change values for intervention vs. control in two studies: − 0.12 bpm, 95% CI: − 2.45 to 2.20, *p* = 0.92 (NS effect in either SB-only or SB + PA intervention). Across the four studies, none found a significant group x time interaction (three included a PA replacement for SB). **CRF measured via METs **[65] NS effect of SB + PA intervention on CRF **CRF measured via exercise capacity (Watts) **[78] Significant effect of SB + PA intervention on CRF	**V̇O**_**2**_**peak** I: 361, C: 278 (8) **Distance** I: 14, C: 29 (1) **Resting HR** I: 65, C: 66 (4) **METs** I: 10, C: 11 (1) **Watts** I1: 23, I2: 22, C: 17 (1)	**Very low certainty** RoB: − 2 points, 6 studies had high RoB, 5 had some concerns Inconsistency: − 1 point, while evidence is mixed, VO_2_ meta-analyses suggest no significant heterogeneity. Indirectness: − 2 points, variation in populations and co-interventions Imprecision: 0 points, OIS met for VO_2_	There is very low certainty of evidence for mixed effects of SB on CRF, but with the potential for SB-focused interventions to improve CRF as evidenced by the VO_2_ meta-analysis. Most SB-only interventions remain underpowered.
Quasi-experimental	**CRF measured via V̇O**_**2**_**peak **[87–89] SB-only: significant intervention effect on CRF with the intervention group experiencing a significant decrease in SB and increase in CRF. SB + PA: One study found NS effect of the intervention on CRF, second study was successful at improving CRF, but unclear if it was effect of reducing SB or increasing PA because of using a desk cycle ergometer. **CRF measured via 6-min walking distance** [84] SB-only: Unclear association between SB and CRF as no formal statistical analysis undertaken. SB appeared to have decreased and CRF increased, but unclear if PA significantly changed. **CRF measured via METs **[82] SB + PA: PA and CRF significantly improved, but no change in SB.	**V̇O**_**2**_**peak** 337 (3) **Distance** 19 (1) **METs** 20 (1)	NA	Evidence from quasi-experimental studies is mixed.
Cohort	**CRF measured via V̇O2peak **[11, 95, 101] All three studies found a significant association between reduced SB and increased CRF (two controlled for PA). One study [101] found a significant association with leisure SB, but not occupational SB. **CRF measured via 6-min walking distance **[92] NS association between change in SB and change in CRF. Study did not control for PA.	**V̇O**_**2**_**peak** 3997 (3) Distance 642 (1)	NA	Evidence from cohort studies generally suggests a significant association between SB and CRF

*C* control group, *CI* confidence interval, *CRF* cardiorespiratory fitness, *HR* heart rate, *I* intervention group, *MET* metabolic equivalents of task, *NA* not applicable, *NS* non-significant, *OIS* optimal information size, *PA* physical activity, *RCT* randomised controlled trial, *RoB* risk of bias, *SB* sedentary behaviour

^a^See Supplementary Tables 9, 11 and 13 for detailed results

**Fig. 3:**
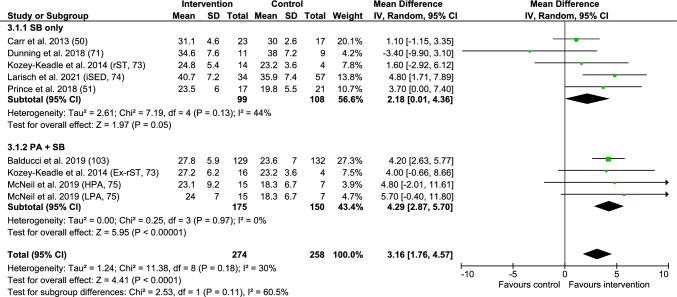
Meta-analysis comparing post-peak oxygen consumption values (mL^.^kg^–1.^min^–1^) in intervention groups with control groups in adults. Note: Prince et al. 2018 included a physical activity intervention (PA) for both the intervention and control group [51]. *CI* confidence interval, *Ex-rST* increase exercise and reduce sedentary time intervention, *HPA* higher-intensity physical activity intervention, *iSED* sedentary intervention, *LPA* lower-intensity physical activity intervention, *rST* reduce sedentary time intervention, *SB* sedentary behaviour intervention, *SD* standard deviation

In adults, seven RCTs [50, 51, 66, 71, 73–75] examined the effects of interventions on V̇O_2_peak. Figure 3 provides the meta-analyses of mean post-value effects for these interventions stratified by whether the intervention arm focused exclusively on reducing/interrupting SB or was included as a component alongside a physical activity intervention. Overall, interventions had a significantly higher post-V̇O_2_peak than control groups (MD = 3.16 mL^.^kg^–1.^ min^–1^, 95% CI: 1.76, 4.57, *p* < 0.0001). There was no statistically significant difference (Chi^2^ = 2.53, df = 1, *p* = 0.11) between interventions for which the primary objective was to reduce SB (MD = 2.18 mL^.^kg^–1.^ min^–1^, 95% CI: 0.01, 4.36) and interventions that targeted increased physical activity (MD = 4.29 mL^.^kg^–1.^ min^–1^, 95% CI: 2.87, 5.70). No statistically significant differences in post-values were observed by a risk of bias (low vs some vs high), population (desk-based workers vs clinical populations), intervention success on reducing SB (reductions in SB statistically significant vs not), SB target (i.e. interrupt vs reduce vs reduce + interrupt), intervention duration (≤ 12 weeks vs > 12 weeks), intervention strategy (i.e. prompts, education, self-monitoring, environment plus prompts, education plus environment, education plus self-monitoring) or V̇O_2_peak measure (direct vs indirect). Sensitivity analyses found that removing the RCT by Dunning et al. [71] caused the overall effect and SB-only intervention effect to increase (MD = 2.67, 95% CI: 0.78, 4.55). The 10-week trial by Dunning et al. sampled young desk-based workers in South Africa and was designed to reduce SB using prompts. While those in the intervention group sat less compared with the control group (4.9 vs 6.4 h/day, *p* = 0.04), there were no significant changes in CRF. Removing the trial by Larisch et al. [74] reduced the SB-focused intervention effects (MD = 1.41, 95% CI: − 0.61, 3.43), and resulted in a significant difference between the SB-focused interventions and those with a physical activity component or focus (Chi^2^ = 5.24, df = 1, *p* = 0.02). The 6-month trial by Larisch et al. sampled middle-aged sedentary office workers in Sweden and was designed to both reduce and interrupt SB using education and environmental and organisational-level changes. The SB intervention arm did not significantly reduce SB.

**Fig. 4:**
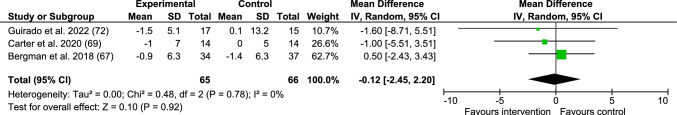
Meta-analysis comparing changes in the resting heart rate in intervention groups with control groups in adults. *CI* confidence interval, *SD* standard deviation

Three adult studies [67, 69, 72] examined the effects of interventions on resting heart rate. Figure 4 provides the meta-analyses of change values for these interventions. Overall, there was no significant effect of SB interventions on changes in the resting heart rate (MD =  − 0.12 bpm, 95% CI: − 2.45, 2.20, *p* = 0.92, I^2^ = 0%, *p* = 0.78).

While meta-analysis data for V̇O_2_peak suggest a significant effect of SB on CRF in adults, evidence across all RCTs is mixed with 11 reporting no statistically significant intervention effect [50, 65, 67–74, 76] and three reporting that CRF increased in the intervention [75, 78, 103] compared with a control group. The quality of evidence from RCTs assessing adults was downgraded to very low because of a risk of bias (six studies had a high risk of bias), inconsistency (estimates of effect were mixed, though a V̇O_2_ meta-analysis suggested no significant heterogeneity) and indirectness (largely office workers and clinical populations and variations in co-interventions and physical activity targets).

### Quasi-Experimental and Cohort Evidence

In youth, evidence from quasi-experimental studies and cohort studies (Tables S10 and S12 of the ESM) was similar to RCT evidence, suggesting mixed effects with some showing statistically significant improvements in CRF with reductions in SB [83, 90, 91, 93, 97, 98, 100, 104] and some showing no effect [85, 86, 94, 99, 102, 105]. In adults, evidence from quasi-experimental studies was mixed (Table S11 of the ESM), with one study showing significant improvements in CRF [87], one study showing no effect [82], and two studies being unclear as to the effect of reduced SB or increased physical activity on CRF [84, 88]. Evidence from cohort studies (Table S13 of the ESM) generally suggested that lower SB is associated with greater CRF, even after controlling for physical activity levels.

## Discussion

This systematic review examined the effects of SB on CRF in youth and adults. Evidence from RCTs suggests mixed associations between SB and CRF, but with the potential for SB to influence CRF as evidenced by results from meta-analyses. In adults, results from the meta-analysis of V̇O_2_peak, which included the most studies and participants, suggest SB interventions could significantly improve CRF. The certainty of evidence was very low, highlighting the need for higher quality RCTs specifically designed and powered to assess this relationship, to improve confidence in the direction and magnitude of effects. Evidence from quasi-experimental studies and cohort studies also suggests mixed associations between SB and CRF.

Previous systematic review evidence for youth was largely based on cross-sectional studies and suggested that SB is negatively associated with CRF [44, 106]. Similarly, previous review evidence from cross-sectional studies involving adults suggested a weak, statistically significant negative association between SB and CRF (r =  − 0.16, 95% CI: − 0.24, − 0.09) [48]. Findings from our review build on the evidence base by providing more prospective and intervention evidence to support benefits.

Several but not all quasi-experimental and cohort studies adjusted for physical activity. Randomised controlled trials are the gold standard for assessing this relationship. Interestingly, out of all the SB-only interventions (i.e. interventions that did not primarily target increased MVPA or did not target the displacement of SB by higher intensity physical activity), only one showed a significant between-group change in MVPA. This finding suggests that improved CRF by increased light-intensity physical activity may be feasible, but the volume of SB reduction may need to be higher than observed. High volumes of SB or prolonged sitting diminish overall skeletal muscle activity and metabolic demand and have deleterious effects on cardiovascular function, including endothelial dysfunction (inability of blood vessels to dilate appropriately), vascular stress, left-ventricular stiffening and blood pressure, which may ultimately be reflected in reduced CRF [107–109]. It is unclear if individuals with sufficient MVPA, but excessive SB, have lower CRF compared with those with sufficient MVPA but low SB.

The RCT by Kozey-Keadle et al. [73] is the only intervention that solely targeted reduced SB and demonstrated a significant improvement in MVPA. Their 12-week RCT assessed whether the combination of aerobic exercise training and reduced SB (EX-rST) resulted in greater improvements in CRF than either reduced SB (rST) or exercise (EX) intervention alone in participants with overweight or obesity. The two groups that targeted reduced SB significantly reduced their SB (r-ST: − 7%, ~ 48 min/day, EX-rST: − 10.3%, ~ 70 min/day). Only the two exercise groups achieved a significant improvement in CRF, possibly suggesting that the addition of exercise MVPA (above incidental physical activity) was likely responsible for the positive changes in CRF. However, these findings and those of our review suggest that SB interventions, which likely have less of an effect on MVPA [110], can maintain and in some cases improve CRF. This is an important finding given the known age-related declines in adult CRF [59, 111, 112], suggesting that incidental/lower intensity physical activity may be sufficient to maintain, but not improve, CRF. This has implications for populations in whom increasing MVPA may be a challenge, but reducing or interrupting SB may be more feasible. Future studies with similar designs of longer duration would help to elucidate the effectiveness of reducing SB in the absence of increases in MVPA.

More recently, there has been a movement to recognise the ‘whole day matters’ with the composition of movement behaviours (e.g. sedentary time, light-intensity physical activity, MVPA, sleep) within a 24-h period having important implications for health across the lifespan. Evidence suggests that SB and light-intensity physical activity, rather than MVPA, are highly correlated and inter-dependent [113]. Compositional isotemporal substitution modelling recognises behaviours are collinear and co-dependent and provides a means of studying how the reallocation of a fixed duration of time spent in one behaviour with another associates with outcomes. While few studies have used isotemporal substitution modelling with fitness as an outcome, there is evidence to suggest that in youth, reallocating activity behaviours to time spent in SB is associated with lower CRF. However, in adults, reallocating any behaviour towards MVPA has been shown to improve CRF [114]. It is important to remember that most of the evidence is cross-sectional, limiting inferences about the temporal nature of relationships.

The sample sizes in most trials were relatively small with CRF often a secondary outcome. Therefore, most studies were likely underpowered (see optimal information size *N* = 134 per group in Table S2 of the ESM). In fact, none of the adult SB-focused RCTs achieved the estimated optimal information size. While many of the individual trial effects may not have been statistically significant, the mean effect sizes suggested improved CRF. The meta-analyses of V̇O_2_peak in adults (MD = 3.16 mL^.^kg^–1.^ min^–1^) suggest that the effects of interventions on CRF are potentially clinically meaningful, with a 1-MET increase (3.5 mL^.^kg^–1.^ min^–1^) associated with reduced risk for all-cause mortality [1, 115]. However, the effects observed in the SB-focused interventions remain below this threshold, though they are consistent with meaningful improvements in clinical populations (e.g. 0.5 MET = 1.75 mL^.^kg^–1.^ min^–1^) [116]. Additionally, a threshold of 1.75 mL^.^kg^–1.^ min^–1^ (0.5 MET) has been considered clinically relevant by some, given a 1-MET change is difficult to achieve for most [117].

### Strengths and Limitations

This review has several strengths including the use of a pre-registered protocol, a comprehensive and peer-reviewed search strategy, the assessment of individual study quality, and the use of a modified GRADE approach to assess the certainty of the evidence and suggest areas of improvement for future studies. In line with previous calls to ascertain the causal associations of SB with outcomes [118], we used a triangulation of evidence (e.g. RCTs, quasi-experiment studies, cohort studies) to obtain a more complete picture of the evidence base. The use of meta-analyses allowed us to combine results from smaller trials; however, the meta-analyses were limited to studies that reported mean changes or post-values using consistent measures and units of CRF. Unfortunately, several of the studies reported adjusted MDs with no post-values or post-values only, with no standard deviations. Furthermore, most of the RCTs were conducted in middle-aged and older adults and targeted sedentary workers or people living with chronic conditions, which increased heterogeneity and limited generalisability. Additionally, most interventions were short in duration (i.e. 12 weeks or less), with few exploring long-term intervention effects (≥ 12 months). While efforts were made to explore sub-group differences, we were limited by the number of included studies.

## Conclusions

Evidence from RCTs suggests mixed associations between SB and CRF, but with the potential for SB to influence CRF as evidenced by results from meta-analyses. Findings from quasi-experimental and cohort studies align with these conclusions. However, further well-designed trials are necessary to validate the relationship, determine the optimal reduction or replacement of SB required to improve CRF, confirm its independence from changes in higher intensity physical activity and explore this relationship in paediatric populations.

### Supplementary Information

Below is the link to the electronic supplementary material.

Supplementary file1 (PDF 521 KB)

Supplementary file2 (PDF 180 KB)

Supplementary file3 (PDF 401 KB)

Supplementary file4 (PDF 303 KB)

Supplementary file5 (PDF 223 KB)

Supplementary file6 (PDF 331 KB)

## Declarations

### Data availability

All data generated or analysed during this systematic review and meta-analysis are included in the article as table(s), figure(s), and/or Online Supplementary Material(s). Any other data requirement can be directed to the corresponding author upon reasonable request.
