# Correction to: The Effect of Sedentary Behaviour on Cardiorespiratory Fitness: A Systematic Review and Meta-Analysis

**DOI:** 10.1007/s40279-024-01995-5

**Published:** 2024-02-12

**Authors:** Stephanie A. Prince, Paddy C. Dempsey, Jennifer L. Reed, Lukas Rubin, Travis J. Saunders, Josephine Ta, Grant R. Tomkinson, Katherine Merucci, Justin J. Lang

**Affiliations:** 1 Centre for Surveillance and Applied Research, Public Health Agency of Canada, 785 Carling Avenue, Ottawa, ON K1A 0K9 Canada; 2 School of Epidemiology and Public Health, Faculty of Medicine, University of Ottawa, Ottawa, ON Canada; 3 Institute for Physical Activity and Nutrition (IPAN), School of Exercise and Nutrition Sciences, Deakin University, Geelong, VIC Australia; 4 MRC Epidemiology Unit, Institute of Metabolic Science, University of Cambridge, Cambridge Biomedical Campus, Cambridge, UK; 5 Diabetes Research Centre, College of Life Sciences, University of Leicester, Leicester, UK; 6 Baker Heart and Diabetes Institute, Melbourne, VIC Australia; 7 Exercise Physiology and Cardiovascular Health Lab, University of Ottawa Heart Institute, Ottawa, ON Canada; 8 School of Human Kinetics, Faculty of Health Sciences, University of Ottawa, Ottawa, ON Canada; 9 Department of Physical Education and Sport, Faculty of Science, Humanities and Education, Technical University of Liberec, Liberec, Czech Republic; 10 Institute of Active Lifestyle, Faculty of Physical Culture, Palacký University Olomouc, Olomouc, Czech Republic; 11 Department Applied Human Sciences, University of Prince Edward Island, Charlottetown, PEI Canada; 12 Telfer School of Management, University of Ottawa, Ottawa, ON Canada; 13 Alliance for Research in Exercise, Nutrition and Activity, Allied Health and Human Performance, University of South Australia, Adelaide, SA Australia; 14 Health Canada Library, Ottawa, ON Canada

**Correction to: Sports Medicine** 10.1007/s40279-023-01986-y

Prince SA, Dempsey PC, Reed JL, Rubin L, Saunders TJ, Ta J, Tomkinson GR, Merucci K, Lang JJ. The effect of sedentary behaviour on cardiorespiratory fitness: a systematic review and meta-analysis. Sports Med. Published on: 16 Jan 2024. 10.1007/s40279-023-01986-y.

In column 2 of Tables 1 and 2 of the above article, the reference numbers provided were incorrect. The tables (now with the correct reference numbers) should have appeared as shown below (Tables 1, 2).

**Table 1:** Summary of findings for sedentary behaviour and cardiorespiratory fitness in youth

Study design	Effect estimates or summary of effect^a^	# of participants (# of studies)	Certainty (quality) of evidence	Interpretation of findings
RCT	**CRF measured via laps completed on the 20-m shuttle run [77, 79, 81]** Pooled mean difference post-values for intervention vs. control: 7.91, 95% CI − 0.65, 16.47, *p* = 0.07 All three studies targeted reduced screen time (two with a PA component [77, 81]). Two studies observed a significant decrease in screen time vs. control [79, 81], the other did not [77]. Only one [81] saw a significant change in PA **CRF measured via recovery HR [80]** NS difference between groups (no difference in PA or SB)	**Laps** I: 618, C: 288 (3) **Recovery HR** I: 60, C: 56 (1)	**Very low certainty** RoB: − 2 points, one study had high RoB, 2 had some concerns Inconsistency: − 1 point, effects from one trial differed, but could likely be explained by intervention target Indirectness: − 2 points, variation in population and co-interventions Imprecision: 0 points, OIS met	There is very low certainty of mixed effects of SB on CRF Note: 3/4 trials targeted PA, 3/4 trials significantly reduced SB
Quasi-experimental studies	**CRF measured via **$\dot{\user2{V}}$**O**_**2**_**peak [83, 85]** One study [83] targeted reduced total SB and found a significant increase in CRF (like the study arms that targeted PA). The other [85] targeted reduced leisure screen time and found that while SB decreased significantly, MVPA also increased significantly and change in CRF was largely correlated with change in MVPA **CRF measured via resting and recovery HR [86]** Both Black and non-Black students saw improvements in their leisure screen time and PA levels. Resting HR only significantly reduced in non-Black students, while recovery HR significantly improved in Black students only	$\dot{\user2{V}}$**O**_**2**_**peak** 120 (2) **Resting/recovery HR** 3813 (1)	NA	Evidence from quasi-experimental studies is mixed (1 positive, 1 mixed, 1 NS)
Cohorts	**CRF measured via **$\dot{\user2{V}}$**O**_**2**_**peak [93, 94, 97, 99, 102]** 3/5 studies (only 1 controlled for PA) observed that reduced screen time or total SB was *not* significantly associated with CRF [94, 99, 102]. Two studies (1 controlled for PA) found that lower screen time was associated with greater CRF [93, 97] **CRF measured via laps completed on the 20-m shuttle run [90, 91, 100, 104, 105]** 3/5 studies (only 1 controlled for PA) observed that reduced SB *was* significantly associated with greater CRF [90, 91, 104]. One study found no significant association [105]. One study found that greater SB was associated with greater CRF [100] **CRF measured via running distance [98]** Evidence suggests a significant association between higher TV watching and reduced CRF	$\dot{\user2{V}}$**O**_**2**_**peak** 4171 (5) **Laps** 4071 (5) **Distance** 135 (1)	NA	Evidence from cohort studies is mixed (6 positive, 4 NS, 1 negative) Three studies controlled for PA in the analysis, and the direction of association differed within each one

*C* control group, *CI* confidence interval, *CRF* cardiorespiratory fitness, *HR* heart rate, *I* intervention group, *METs* metabolic equivalents of task, *NA* not applicable, *OIS* optimal information size, *PA* physical activity, *RCT* randomized controlled trial, *RoB* risk of bias, *SB* sedentary behaviour, *TV* television

^a^See supplementary Tables S8, S10 and S12 of the ESM for detailed results

**Table 2:** Summary of findings for sedentary behaviour and cardiorespiratory fitness in adults

Study design	Effect estimates or summary of effect	# of participants (# of studies)	Certainty (quality) of evidence	Interpretation of findings
RCT^a^	**CRF measured via **$\dot{\user2{V}}$**O**_**2**_**peak [50, 51, 71, 73, 75, 76, 103]** Pooled mean difference post-values for $\dot{V}$O_2_peak intervention vs. control: 3.16 mL⋅kg^−1^⋅min^−1^, 95% CI 1.76 to 4.57, *p* < 0.00001 SB-only: 2.18, 95% CI 0.01 to 4.36, *p* = 0.05 SB + PA: 4.29, 95% CI 2.87 to 5.70, *p* < 0.00001 **CRF measured via running distance [70]** NS effect of SB-only intervention on CRF **CRF measured via resting HR [67–69, 72]** Pooled mean difference change values for intervention vs. control in two studies: − 0.12 bpm, 95% CI − 2.45 to 2.20, *p* = 0.92 (NS effect in either SB-only or SB + PA intervention). Across the four studies, none found a significant group x time interaction (three included a PA replacement for SB) **CRF measured via METs [65]** NS effect of SB + PA intervention on CRF **CRF measured via exercise capacity (Watts) [78]** Significant effect of SB + PA intervention on CRF	$\dot{\user2{V}}$**O**_**2**_**peak** I: 361, C: 278 (8) **Distance** I: 14, C: 29 (1) **Resting HR** I: 65, C: 66 (4) **METs** I: 10, C: 11 (1) **Watts** I1: 23, I2: 22, C: 17 (1)	**Very low certainty** RoB: − 2 points, 6 studies had high RoB, 5 had some concerns Inconsistency: − 1 point, while evidence is mixed, *V*O_2_ meta-analyses suggest no significant heterogeneity Indirectness: − 2 points, variation in populations and co-interventions Imprecision: 0 points, OIS met for *V*O_2_	There is very low certainty of evidence for mixed effects of SB on CRF, but with the potential for SB-focused interventions to improve CRF as evidenced by the *V*O_2_ meta-analysis. Most SB-only interventions remain underpowered
Quasi-experimental	**CRF measured via **$\dot{\user2{V}}$**O**_**2**_**peak [87–89]** SB-only: significant intervention effect on CRF with the intervention group experiencing a significant decrease in SB and increase in CRF SB + PA: One study found NS effect of the intervention on CRF, second study was successful at improving CRF, but unclear if it was effect of reducing SB or increasing PA because of using a desk cycle ergometer **CRF measured via 6-min walking distance [84]** SB-only: Unclear association between SB and CRF as no formal statistical analysis undertaken. SB appeared to have decreased and CRF increased, but unclear if PA significantly changed **CRF measured via METs [82]** SB + PA: PA and CRF significantly improved, but no change in SB	$\dot{\user2{V}}$**O**_**2**_**peak** 337 (3) **Distance** 19 (1) **METs** 20 (1)	NA	Evidence from quasi-experimental studies is mixed
Cohort	**CRF measured via **$\dot{\user2{V}}$**O**_**2**_**peak [11, 95, 101]** All three studies found a significant association between reduced SB and increased CRF (two controlled for PA). One study [101] found a significant association with leisure SB, but not occupational SB **CRF measured via 6-min walking distance [92]** NS association between change in SB and change in CRF. Study did not control for PA	$\dot{\user2{V}}$**O**_**2**_**peak** 3997 (3) Distance 642 (1)	NA	Evidence from cohort studies generally suggests a significant association between SB and CRF

*C* control group, *CI* confidence interval, *CRF* cardiorespiratory fitness, *HR* heart rate, *I* intervention group, *METs* metabolic equivalents of task, *NA* not applicable, *OIS* optimal information size, *PA* physical activity, *RCT* randomized controlled trial, *RoB* risk of bias, *SB* sedentary behaviour

^a^See Supplementary Tables S9, S11 and S13 of the ESM for detailed results

