# Supplementary material for: The Effect of Sedentary Behaviour on Cardiorespiratory Fitness: A Systematic Review and Meta-Analysis
Source: Sports Med. 2024 Jan 16;54(4):997–1013. doi: 10.1007/s40279-023-01986-y (PMC11052788; doi:10.1007/s40279-023-01986-y)
Supplement: Supplementary file 1 — Supplementary file1 (PDF 521 KB) [file 40279_2023_1986_MOESM1_ESM.pdf]

Title: The effect of sedentary behaviour on cardiorespiratory fitness: a systematic review and meta-analysis.
Journal: Sports Medicine
Authors: Stephanie A. Prince*, Paddy C. Dempsey, Jennifer L. Reed, Lukas Rubin, Travis J. Saunders, Josephine Ta, Grant R. Tomkinson, Katherine Merucci, Justin J. Lang
***Corresponding author:** Centre for Surveillance and Applied Research, Public Health Agency of Canada, stephanie.prince.ware@phac-aspc.gc.ca

Table S1 – Search strategies

Medline

Database(s): **Ovid MEDLINE(R) ALL** 1946 to August 26, 2022
Search Strategy:

#	Searches	Results
1	*Sedentary Behavior/ or *sitting position/ or *screen time/ or *video games/ or *television/ or *"internet use"/ or exp *computers, handheld/	28864
2	(sedentar* or stationary or stationery).ti,kf.	19406
3	(sedentar* or stationary or stationery).ab. /freq=2	28245
4	(sedentary behavio?r and sb).ab.	1041
5	(physical* adj3 inactiv*).ti,kf.	1708
6	(physical* adj3 inactiv*).ab. /freq=2	2593
7	(sitting or sit or sits or seated or recline? or reclining or lying or "lie down" or "lay down" or "laying down" or deskbound or (bound adj (desk? or chair?))).ti,kf.	11037
8	(sitting or sit or sits or seated or recline? or reclining or lying or "lie down" or "lay down" or "laying down" or deskbound or (bound adj (desk? or chair?))).ab. /freq=2	22010
9	(screen? adj2 (time or watch* or view*)).ti,kf. or (screen? adj2 (time or watch* or view*)).ab. /freq=2	2655
10	(video? adj2 (stream* or watch* or time or view*)).ti,kf. or (video? adj2 (stream* or watch* or time or view*)).ab. /freq=2	1619
11	(television? or tv or "t.v.").ti,kf. or (television? or tv or "t.v.").ab. /freq=2	15922
12	(((apple or samsung or android or amazon or google or computer? or time or watch* or view* or play* or stream* or game? or gaming) adj3 tablet?) or ipad?).ti,kf. or (((apple or samsung or android or amazon or google or computer? or time or watch* or view* or play* or stream* or game? or gaming) adj3 tablet?) or ipad?).ab. /freq=2	1566

13	((brows* or stream* or surf* or time or use? or using or watch*) adj3 (internet or web* or net)).ti,kf. or ((brows* or stream* or surf* or time or use? or using or watch*) adj3 (internet or web* or net)).ab. /freq=2	9893
14	(((video or computer or console) adj game?) or videogam* or gaming).ti,kf. or (((video or computer or console) adj game?) or videogam* or gaming).ab. /freq=2	6516
15	or/1-14 [SEDENTARY BEHAVIOUR]	114715
16	*Cardiorespiratory fitness/	2199
17	((cardiorespirator* or cardio respirator* or aerobic or cardiopulmonar* or cardio pulmonar* or cardiovascular* or cardio vascular* or physical* work* or cardiometaboli* or cardio metaboli*) adj4 (fitness or capacit* or endurance or perform* or health*)).ti,kf. or ((cardiorespirator* or cardio respirator* or aerobic or cardiopulmonar* or cardio pulmonar* or cardiovascular* or cardio vascular* or physical* work* or cardiometaboli* or cardio metaboli*) adj4 (fitness or capacit* or endurance or perform* or health*)).ab. /freq=2	24622
18	(Cardiorespiratory fitness and CRF).ab.	1567
19	(Cardiovascular Health Study and CHS).ab.	401
20	exp *exercise tests/	20948
21	((minute or mile or distance or timed) adj3 run).ti,kf. or ((minute or mile or distance or timed) adj3 run).ab. /freq=2	401
22	((fitness or exercise or endurance or step or walk or run or beep or tread?mill or ergometry or eurofit or stress) adj3 test*).ti,kf. or ((fitness or exercise or endurance or step or walk or run or beep or tread?mill or ergometry or eurofit or stress) adj3 test*).ab. /freq=2	32954
23	((functional or aerobic or exercise) adj3 (capacity or endurance)).ti,kf. or ((functional or aerobic or exercise) adj3 (capacity or endurance)).ab. /freq=2	21290
24	((Submaximal or maximal or graded) adj3 (treadmill? or tread mill? or ergometer?)).ti,ab,kf.	3672
25	(maxim* oxygen or peak oxygen or VO2*).ti,kf. or (maxim* oxygen or peak oxygen or VO2*).ab. /freq=2	24260
26	or/16-25 [CARDIOVASCULAR HEALTH]	98172
27	15 and 26 [SEDENTARY BEHAVIOUR + CARDIOVASCULAR HEALTH]	3692
28	*pregnancy/ or *pregnant women/	38276
29	(pregnan* or gestation* or primigravida? or multigravida? or secundigravida?).ti.	288378

30	28 or 29	298947
31	(nonpregnan* or non pregnan*).ti.	3106
32	30 not 31 [PREGNANCY]	296443
33	27 not 32 [SEDENTARY BEHAVIOUR + CARDIOVASCULAR HEALTH + PREGNANCY EXCLUDED]	3672
34	randomized controlled trial.pt.	575762
35	controlled clinical trial.pt.	95006
36	Randomized controlled trial/	575762
37	Double-Blind Method/	172859
38	Single-Blind Method/	32147
39	randomi#ed.ti,ab,kf.	743973
40	(allocat* adj3 (random or randomly)).ti,ab,kf.	40431
41	trial?.ti,ab,kf.	1235176
42	((singl* or doubl* or tripl* or trebl*) adj (blind* or dumm* or mask*)).ti,ab,kf.	191180
43	or/34-42	1737112
44	(exp animals/ or exp animal experimentation/ or exp models, animal/) not (humans/ or exp human experimentation/ or exp persons/ or human*.ti.)	5018740
45	(animal* or ape or apes or chimpanzee* or gerbil* or guineapig* or guinea pig* or hamster? or hare or hares or macaque* or mammal* or mice or monkey* or mouse or primate* or rabbit* or rat or rats or rodent?).ti.	1919847
46	44 or 45	5403811
47	43 not 46 [RCT filter adapted from "Technical Supplement to Chapter 4: Searching for and Selecting Studies" in *Cochrane Handbook for Systematic Reviews of Interventions* Version 6 (2019)]	1629151
48	33 and 47 [FINAL SET LIMITED TO RCTs]	952
49	33 not 48	2720
50	49 not 46 [FINAL SET - ALL REMAINING STUDIES]	2464

Embase

Database(s): **Embase** 1974 to 2022 August 26
Search Strategy:

#	Searches	Results

1	*sedentary lifestyle/ or *sedentary time/ or *sitting/ or exp *video game/ or exp *screen time/ or *"internet use"/ or *television/ or exp *television viewing/ or *smartphone/ or exp *personal computer/	29851
2	(sedentar* or stationary or stationery).ti,kw.	21593
3	(sedentar* or stationary or stationery).ab. /freq=2	33833
4	(sedentary behavio?r and sb).ab.	1154
5	(physical* adj3 inactiv*).ti,kw.	1756
6	(physical* adj3 inactiv*).ab. /freq=2	3401
7	(sitting or sit or sits or seated or recline? or reclining or lying or "lie down" or "lay down" or "laying down" or deskbound or (bound adj (desk? or chair?))).ti,kw.	10805
8	(sitting or sit or sits or seated or recline? or reclining or lying or "lie down" or "lay down" or "laying down" or deskbound or (bound adj (desk? or chair?))).ab. /freq=2	28953
9	(screen? adj2 (time or watch* or view*)).ti,kw. or (screen? adj2 (time or watch* or view*)).ab. /freq=2	2721
10	(video? adj2 (stream* or watch* or time or view*)).ti,kw. or (video? adj2 (stream* or watch* or time or view*)).ab. /freq=2	2145
11	(television? or tv or "t.v.").ti,kw. or (television? or tv or "t.v.").ab. /freq=2	19999
12	(((apple or samsung or android or amazon or google or computer? or time or watch* or view* or play* or stream* or game? or gaming) adj3 tablet?) or ipad?).ti,kw. or (((apple or samsung or android or amazon or google or computer? or time or watch* or view* or play* or stream* or game? or gaming) adj3 tablet?) or ipad?).ab. /freq=2	2463
13	((brows* or stream* or surf* or time or use? or using or watch*) adj3 (internet or web* or net)).ti,kw. or ((brows* or stream* or surf* or time or use? or using or watch*) adj3 (internet or web* or net)).ab. /freq=2	12269
14	(((video or computer or console) adj game?) or videogam* or gaming).ti,kw. or (((video or computer or console) adj game?) or videogam* or gaming).ab. /freq=2	7015
15	or/1-14 [SEDENTARY BEHAVIOUR]	137735
16	*Cardiorespiratory fitness/	3215
17	((cardiorespirator* or cardio respirator* or aerobic or cardiopulmonar* or cardio pulmonar* or cardiovascular* or cardio vascular* or physical* work* or cardiometaboli* or cardio metaboli*) adj4 (fitness or capacit* or endurance or perform* or health*)).ti,kw. or ((cardiorespirator* or cardio respirator* or aerobic or cardiopulmonar* or cardio pulmonar* or cardiovascular* or cardio vascular* or	31967

	physical* work* or cardiometaboli* or cardio metaboli*) adj4 (fitness or capacit* or endurance or perform* or health*)).ab. /freq=2	
18	(Cardiorespiratory fitness and CRF).ab.	2042
19	(Cardiovascular Health Study and CHS).ab.	633
20	exp *exercise test/	23645
21	((minute or mile or distance or timed) adj3 run).ti,kw. or ((minute or mile or distance or timed) adj3 run).ab. /freq=2	449
22	((fitness or exercise or endurance or step or walk or run or beep or tread?mill or ergometry or eurofit or stress) adj3 test*).ti,kw. or ((fitness or exercise or endurance or step or walk or run or beep or tread?mill or ergometry or eurofit or stress) adj3 test*).ab. /freq=2	43995
23	((functional or aerobic or exercise) adj3 (capacity or endurance)).ti,kw. or ((functional or aerobic or exercise) adj3 (capacity or endurance)).ab. /freq=2	29670
24	((Submaximal or maximal or graded) adj3 (treadmill? or tread mill? or ergometer?)).ti,ab,kw.	4493
25	(maxim* oxygen or peak oxygen or VO2*).ti,kw. or (maxim* oxygen or peak oxygen or VO2*).ab. /freq=2	33194
26	or/16-25 [CARDIOVASCULAR HEALTH]	128403
27	15 and 26 [SEDENTARY BEHAVIOUR + CARDIOVASCULAR HEALTH]	4787
28	(pregnan* or gestation* or primigravida? or multigravida? or secundigravida?).ti.	330387
29	(nonpregnan* or non pregnan*).ti.	3202
30	28 not 29 [PREGNANCY]	327765
31	27 not 30 [SEDENTARY BEHAVIOUR + CARDIOVASCULAR HEALTH + PREGNANCY EXCLUDED]	4770
32	randomized controlled trial/	723872
33	controlled clinical trial/	466887
34	randomi#ed.ti,ab.	1061229
35	(allocat* adj3 (random or randomly)).ti,ab.	49591
36	randomization/	94663
37	intermethod comparison/	286704
38	placebo.ti,ab.	345308
39	(compare or compared or comparison).ti.	572529

40	((evaluated or evaluate or evaluating or assessed or assess) and (compare or compared or comparing or comparison)).ab.	2554323
41	(open adj1 label).ti,ab.	99095
42	((singl* or doubl* or tripl* or trebl*) adj (blind* or dumm* or mask*)).ti,ab.	267178
43	double blind procedure/	197939
44	(parallel adj1 group*).ti,ab.	30428
45	cross?over.ti,ab.	84070
46	((assign* or match or matched or allocation) adj6 (alternate or group or groups or intervention or interventions or patient or patients or subject or subjects or participant or participants)).ti,ab.	427263
47	(assigned or allocated).ti,ab.	455670
48	(controlled adj8 (study or design or trial)).ti,ab.	422988
49	(volunteer or volunteers).ti,ab.	270628
50	trial.ti.	367033
51	or/32-50	5032004
52	(random* sampl* adj8 (cross section* or questionnaire* or survey or surveys or database or databases)).ti,ab. not (comparative study/ or controlled study/ or (randomised controlled or randomized controlled or randomly assigned).ti,ab.)	9552
53	"cross-sectional study"/ not (randomized controlled trial/ or controlled clinical study/ or controlled study/ or (randomised controlled or randomized controlled or control group or control groups).ti,ab.)	3
54	((case control* and random*) not (randomised controlled or randomized controlled)).ti,ab.	20169
55	(systematic review not (trial or study)).ti.	219766
56	(nonrandom* not random*).ti,ab.	18000
57	random field*.ti,ab.	2768
58	(random cluster adj4 sampl*).ti,ab.	1471
59	(review.ab. and review.pt.) not trial.ti.	1018767
60	"we searched".ab. and review.ti,pt.	43311
61	update review.ab.	120
62	(databases adj5 searched).ab.	56946

63	(rat or rats or mouse or mice or swine or porcine or murine or sheep or lambs or pigs or piglets or rabbit or rabbits or cat or cats or dog or dogs or cattle or bovine or monkey or monkeys or trout or marmoset*).ti. and animal experiment/	1162868
64	animal experiment/ not (human experiment/ or human/)	2440704
65	or/52-64	3723548
66	51 not 65 [RCT filter adapted from "Technical Supplement to Chapter 4: Searching for and Selecting Studies" in *Cochrane Handbook for Systematic Reviews of Interventions* Version 6 (2019)]	4520886
67	31 and 66	1836
68	conference abstract.pt.	4509958
69	67 not 68 [FINAL SET LIMITED TO RCTs]	1377
70	31 not (63 or 64 or 68 or 69) [FINAL SET - ALL REMAINING STUDIES]	1934

Scopus – RCTs

1646 Results

((((TITLE (sedentar* OR stationary OR stationery) OR KEY (sedentar* OR stationary OR stationery) OR ABS (("sedentary behavior" OR "sedentary behaviour") AND "sb") OR TITLE (physical* W/3 inactiv*) OR KEY (physical* W/3 inactiv*) OR TITLE (sitting OR sit OR sits OR seated OR recline* OR reclining OR lying OR "lie down" OR "lay down" OR "laying down" OR deskbound OR (bound W/1 (desk* OR chair*))) OR KEY (sitting OR sit OR sits OR seated OR recline* OR reclining OR lying OR "lie down" OR "lay down" OR "laying down" OR deskbound OR (bound W/1 (desk* OR chair*))) OR TITLE (screen* W/2 (time OR watch* OR view*)) OR KEY (screen* W/2 (time OR watch* OR view*)) OR TITLE (video* W/2 (stream* OR watch* OR time OR view*)) OR KEY (video* W/2 (stream* OR watch* OR time OR view*)) OR TITLE (television* OR tv OR "t.v.") OR KEY (television* OR tv OR "t.v.") OR TITLE (((apple OR samsung OR android OR amazon OR google OR computer* OR time OR watch* OR view* OR play* OR stream* OR game* OR gaming) W/3 tablet*) OR ipad*) OR KEY (((apple OR samsung OR android OR amazon OR google OR computer* OR time OR watch* OR view* OR play* OR stream* OR game* OR gaming) W/3 tablet*) OR ipad*) OR TITLE ((brows* OR stream* OR surf* OR time OR use* OR using OR watch*) W/3 (internet OR web* OR net)) OR KEY ((brows* OR stream* OR surf* OR time OR use* OR using OR watch*) W/3 (internet OR web* OR net)) OR TITLE (((video OR computer OR console) W/1 game*) OR videogam* OR gaming) OR KEY (((video OR computer OR console) W/1 game*) OR videogam* OR gaming)) AND (TITLE ((cardiorespirator* OR "cardio respirator*" OR aerobic OR cardiopulmonar* OR "cardio pulmonar*" OR cardiovascular* OR "cardio vascular*" OR "physical* work*" OR cardiometaboli* OR "cardio metaboli*") W/4 (fitness OR capacit* OR endurance OR perform* OR health*)) OR KEY ((cardiorespirator* OR "cardio respirator*" OR aerobic OR cardiopulmonar* OR "cardio

pulmonar*" OR cardiovascular* OR "cardio vascular*" OR "physical* work*" OR cardiometaboli* OR "cardio metaboli*") W/4 (fitness OR capacit* OR endurance OR perform* OR health*)) OR ABS ("Cardiorespiratory fitness" AND "CRF") OR ABS ("Cardiovascular Health Study" AND "CHS") OR TITLE ((minute OR mile OR distance OR timed) W/3 run) OR ABS ((minute OR mile OR distance OR timed) W/3 run) OR TITLE ((fitness OR exercise OR endurance OR step OR walk OR run OR beep OR "tread*mill" OR ergometry OR eurofit OR stress) W/3 test*) OR ABS ((fitness OR exercise OR endurance OR step OR walk OR run OR beep OR "tread*mill" OR ergometry OR eurofit OR stress) W/3 test*) OR TITLE ((functional OR aerobic OR exercise) W/3 (capacity OR endurance)) OR KEY ((functional OR aerobic OR exercise) W/3 (capacity OR endurance)) OR TITLE-ABS-KEY ((submaximal OR maximal OR graded) W/3 (treadmill* OR "tread mill*" OR ergometer*)) OR TITLE ("maxim* oxygen" OR "peak oxygen" OR vo2*) OR KEY ("maxim* oxygen" OR "peak oxygen" OR vo2*))) AND NOT (TITLE ((pregnan* OR gestation* OR primigravida* OR multigravida* OR secundigravida*) AND NOT (nonpregnan* OR "non pregnan*")))) AND NOT ((INDEXTERMS (animals OR "animal experimentation" OR "models, animal" OR "animal experiment") AND NOT (INDEXTERMS (humans OR "human experimentation" OR persons OR "human experiment") OR TITLE (human*))) OR TITLE ({rat} OR {rats} OR mouse OR mice OR swine OR porcine OR murine OR sheep OR lambs OR pigs OR piglets OR rabbit OR rabbits OR {cat} OR cats OR {dog} OR dogs OR cattle OR bovine OR monkey OR monkeys OR trout OR marmoset* OR animal*) OR DOCTYPE (cp))) AND ((INDEXTERMS ("randomized controlled trial" OR "double-blind method" OR "single-blind method" OR "controlled clinical trial" OR "randomization" OR "intermethod comparison" OR "double blind procedure") OR TITLE-ABS-KEY (randomized OR randomised) OR TITLE-ABS-KEY (allocat* W/3 (random OR randomly)) OR TITLE (trial OR trials) OR TITLE-ABS-KEY ((singl* OR doubl* OR tripl* OR trebl*) PRE/0 (blind* OR dumm* OR mask*)) OR TITLE-ABS (placebo) OR TITLE (compare OR compared OR comparison) OR ABS ((evaluated OR evaluate OR evaluating OR assessed OR assess) AND (compare OR compared OR comparing OR comparison)) OR TITLE-ABS (open W/1 label) OR TITLE-ABS (parallel W/1 group*) OR TITLE-ABS (crossover OR "cross over") OR TITLE-ABS ((assign* OR match OR matched OR allocation) W/6 (alternate OR group OR groups OR intervention OR interventions OR patient OR patients OR subject OR subjects OR participant OR participants)) OR TITLE-ABS (assigned OR allocated) OR TITLE-ABS (controlled W/8 (study OR design OR trial)) OR TITLE-ABS (volunteer OR volunteers)) AND NOT ((TITLE-ABS ("random* sampl*" W/8 ("cross section*" OR questionnaire* OR survey OR surveys OR database OR databases)) AND NOT (INDEXTERMS ("comparative study") OR INDEXTERMS ("controlled study") OR TITLE-ABS ("randomised controlled" OR "randomized controlled" OR "randomly assigned"))) OR (INDEXTERMS ("cross-sectional study") AND NOT (INDEXTERMS ("randomized controlled trial") OR INDEXTERMS ("controlled clinical study") OR INDEXTERMS ("controlled study") OR TITLE-ABS ("randomised controlled" OR "randomized controlled" OR "control group" OR "control groups"))) OR (TITLE-ABS (("case control*" AND random*) AND NOT ("randomised controlled" OR "randomized controlled"))) OR (TITLE ("systematic review" AND NOT (trial OR study))) OR (TITLE-ABS (nonrandom* AND NOT random*)) OR (TITLE-ABS ("random field*")) OR (TITLE-ABS ("random cluster" W/4 sampl*)) OR ((ABS (review) AND DOCTYPE (re)) AND NOT TITLE (trial)) OR (ABS ("we searched") AND DOCTYPE (re)) OR (ABS ("update review")) OR (ABS (databases W/5 searched)))))

Scopus - Remaining Studies

2629 Results

((((TITLE (sedentar* OR stationary OR stationery) OR KEY (sedentar* OR stationary OR stationery) OR ABS (("sedentary behavior" OR "sedentary behaviour") AND "sb") OR TITLE (physical* W/3 inactiv*) OR KEY (physical* W/3 inactiv*) OR TITLE (sitting OR sit OR sits OR seated OR recline* OR reclining OR lying OR "lie down" OR "lay down" OR "laying down" OR deskbound OR (bound W/1 (desk* OR chair*))) OR KEY (sitting OR sit OR sits OR seated OR recline* OR reclining OR lying OR "lie down" OR "lay down" OR "laying down" OR deskbound OR (bound W/1 (desk* OR chair*))) OR TITLE (screen* W/2 (time OR watch* OR view*)) OR KEY (screen* W/2 (time OR watch* OR view*)) OR TITLE (video* W/2 (stream* OR watch* OR time OR view*)) OR KEY (video* W/2 (stream* OR watch* OR time OR view*)) OR TITLE (television* OR tv OR "t.v.") OR KEY (television* OR tv OR "t.v.") OR TITLE (((apple OR samsung OR android OR amazon OR google OR computer* OR time OR watch* OR view* OR play* OR stream* OR game* OR gaming) W/3 tablet*) OR ipad*) OR KEY (((apple OR samsung OR android OR amazon OR google OR computer* OR time OR watch* OR view* OR play* OR stream* OR game* OR gaming) W/3 tablet*) OR ipad*) OR TITLE ((brows* OR stream* OR surf* OR time OR use* OR using OR watch*) W/3 (internet OR web* OR net)) OR KEY ((brows* OR stream* OR surf* OR time OR use* OR using OR watch*) W/3 (internet OR web* OR net)) OR TITLE (((video OR computer OR console) W/1 game*) OR videogam* OR gaming) OR KEY (((video OR computer OR console) W/1 game*) OR videogam* OR gaming)) AND (TITLE ((cardiorespirator* OR "cardio respirator*" OR aerobic OR cardiopulmonar* OR "cardio pulmonar*" OR cardiovascular* OR "cardio vascular*" OR "physical* work*" OR cardiometaboli* OR "cardio metaboli*") W/4 (fitness OR capacit* OR endurance OR perform* OR health*)) OR KEY ((cardiorespirator* OR "cardio respirator*" OR aerobic OR cardiopulmonar* OR "cardio pulmonar*" OR cardiovascular* OR "cardio vascular*" OR "physical* work*" OR cardiometaboli* OR "cardio metaboli*") W/4 (fitness OR capacit* OR endurance OR perform* OR health*)) OR ABS ("Cardiorespiratory fitness" AND "CRF") OR ABS ("Cardiovascular Health Study" AND "CHS") OR TITLE ((minute OR mile OR distance OR timed) W/3 run) OR ABS ((minute OR mile OR distance OR timed) W/3 run) OR TITLE ((fitness OR exercise OR endurance OR step OR walk OR run OR beep OR "tread*mill" OR ergometry OR eurofit OR stress) W/3 test*) OR ABS ((fitness OR exercise OR endurance OR step OR walk OR run OR beep OR "tread*mill" OR ergometry OR eurofit OR stress) W/3 test*) OR TITLE ((functional OR aerobic OR exercise) W/3 (capacity OR endurance)) OR KEY ((functional OR aerobic OR exercise) W/3 (capacity OR endurance)) OR TITLE-ABS-KEY ((submaximal OR maximal OR graded) W/3 (treadmill* OR "tread mill*" OR ergometer*)) OR TITLE ("maxim* oxygen" OR "peak oxygen" OR vo2*) OR KEY ("maxim* oxygen" OR "peak oxygen" OR vo2*))) AND NOT (TITLE ((pregnan* OR gestation* OR primigravida* OR multigravida* OR secundigravida*) AND NOT (nonpregnan* OR "non pregnan*")))) AND NOT ((INDEXTERMS (animals OR "animal experimentation" OR "models, animal" OR "animal experiment") AND NOT (INDEXTERMS (humans OR "human experimentation" OR persons OR "human experiment") OR TITLE (human*))) OR TITLE ({rat} OR {rats} OR mouse OR mice OR

swine OR porcine OR murine OR sheep OR lambs OR pigs OR piglets OR rabbit OR rabbits OR {cat} OR cats OR {dog} OR dogs OR cattle OR bovine OR monkey OR monkeys OR trout OR marmoset* OR animal*) OR DOCTYPE (cp))) AND NOT (((((TITLE (sedentar* OR stationary OR stationery) OR KEY (sedentar* OR stationary OR stationery) OR ABS (("sedentary behavior" OR "sedentary behaviour") AND "sb") OR TITLE (physical* W/3 inactiv*) OR KEY (physical* W/3 inactiv*) OR TITLE (sitting OR sit OR sits OR seated OR recline* OR reclining OR lying OR "lie down" OR "lay down" OR "laying down" OR deskbound OR (bound W/1 (desk* OR chair*))) OR KEY (sitting OR sit OR sits OR seated OR recline* OR reclining OR lying OR "lie down" OR "lay down" OR "laying down" OR deskbound OR (bound W/1 (desk* OR chair*))) OR TITLE (screen* W/2 (time OR watch* OR view*)) OR KEY (screen* W/2 (time OR watch* OR view*)) OR TITLE (video* W/2 (stream* OR watch* OR time OR view*)) OR KEY (video* W/2 (stream* OR watch* OR time OR view*)) OR TITLE (television* OR tv OR "t.v.") OR KEY (television* OR tv OR "t.v.") OR TITLE (((apple OR samsung OR android OR amazon OR google OR computer* OR time OR watch* OR view* OR play* OR stream* OR game* OR gaming) W/3 tablet*) OR ipad*) OR KEY (((apple OR samsung OR android OR amazon OR google OR computer* OR time OR watch* OR view* OR play* OR stream* OR game* OR gaming) W/3 tablet*) OR ipad*) OR TITLE ((brows* OR stream* OR surf* OR time OR use* OR using OR watch*) W/3 (internet OR web* OR net)) OR KEY ((brows* OR stream* OR surf* OR time OR use* OR using OR watch*) W/3 (internet OR web* OR net)) OR TITLE (((video OR computer OR console) W/1 game*) OR videogam* OR gaming) OR KEY (((video OR computer OR console) W/1 game*) OR videogam* OR gaming)) AND (TITLE ((cardiorespirator* OR "cardio respirator*" OR aerobic OR cardiopulmonar* OR "cardio pulmonar*" OR cardiovascular* OR "cardio vascular*" OR "physical* work*" OR cardiometaboli* OR "cardio metaboli*") W/4 (fitness OR capacit* OR endurance OR perform* OR health*)) OR KEY ((cardiorespirator* OR "cardio respirator*" OR aerobic OR cardiopulmonar* OR "cardio pulmonar*" OR cardiovascular* OR "cardio vascular*" OR "physical* work*" OR cardiometaboli* OR "cardio metaboli*") W/4 (fitness OR capacit* OR endurance OR perform* OR health*)) OR ABS ("Cardiorespiratory fitness" AND "CRF") OR ABS ("Cardiovascular Health Study" AND "CHS") OR TITLE ((minute OR mile OR distance OR timed) W/3 run) OR ABS ((minute OR mile OR distance OR timed) W/3 run) OR TITLE ((fitness OR exercise OR endurance OR step OR walk OR run OR beep OR "tread*mill" OR ergometry OR eurofit OR stress) W/3 test*) OR ABS ((fitness OR exercise OR endurance OR step OR walk OR run OR beep OR "tread*mill" OR ergometry OR eurofit OR stress) W/3 test*) OR TITLE ((functional OR aerobic OR exercise) W/3 (capacity OR endurance)) OR KEY ((functional OR aerobic OR exercise) W/3 (capacity OR endurance)) OR TITLE-ABS-KEY ((submaximal OR maximal OR graded) W/3 (treadmill* OR "tread mill*" OR ergometer*)) OR TITLE ("maxim* oxygen" OR "peak oxygen" OR vo2*) OR KEY ("maxim* oxygen" OR "peak oxygen" OR vo2*))) AND NOT (TITLE ((pregnan* OR gestation* OR primigravida* OR multigravida* OR secundigravida*) AND NOT (nonpregnan* OR "non pregnan*")))) AND NOT ((INDEXTERMS (animals OR "animal experimentation" OR "models, animal" OR "animal experiment") AND NOT (INDEXTERMS (humans OR "human experimentation" OR persons OR "human experiment") OR TITLE (human*))) OR TITLE ({rat} OR {rats} OR mouse OR mice OR swine OR porcine OR murine OR sheep OR lambs OR pigs OR piglets OR rabbit OR rabbits OR {cat} OR cats OR {dog} OR dogs OR cattle OR

bovine OR monkey OR monkeys OR trout OR marmoset* OR animal*) OR DOCTYPE (cp))) AND ((INDEXTERMS ("randomized controlled trial" OR "double-blind method" OR "single-blind method" OR "controlled clinical trial" OR "randomization" OR "intermethod comparison" OR "double blind procedure") OR TITLE-ABS-KEY (randomized OR randomised) OR TITLE-ABS-KEY (allocat* W/3 (random OR randomly)) OR TITLE (trial OR trials) OR TITLE-ABS-KEY ((singl* OR doubl* OR tripl* OR trebl*) PRE/0 (blind* OR dumm* OR mask*)) OR TITLE-ABS (placebo) OR TITLE (compare OR compared OR comparison) OR ABS ((evaluated OR evaluate OR evaluating OR assessed OR assess) AND (compare OR compared OR comparing OR comparison)) OR TITLE-ABS (open W/1 label) OR TITLE-ABS (parallel W/1 group*) OR TITLE-ABS (crossover OR "cross over") OR TITLE-ABS ((assign* OR match OR matched OR allocation) W/6 (alternate OR group OR groups OR intervention OR interventions OR patient OR patients OR subject OR subjects OR participant OR participants)) OR TITLE-ABS (assigned OR allocated) OR TITLE-ABS (controlled W/8 (study OR design OR trial)) OR TITLE-ABS (volunteer OR volunteers)) AND NOT ((TITLE-ABS ("random* sampl*" W/8 ("cross section*" OR questionnaire* OR survey OR surveys OR database OR databases)) AND NOT (INDEXTERMS ("comparative study") OR INDEXTERMS ("controlled study") OR TITLE-ABS ("randomised controlled" OR "randomized controlled" OR "randomly assigned"))) OR (INDEXTERMS ("cross-sectional study") AND NOT (INDEXTERMS ("randomized controlled trial") OR INDEXTERMS ("controlled clinical study") OR INDEXTERMS ("controlled study") OR TITLE-ABS ("randomised controlled" OR "randomized controlled" OR "control group" OR "control groups"))) OR (TITLE-ABS (("case control*" AND random*) AND NOT ("randomised controlled" OR "randomized controlled"))) OR (TITLE ("systematic review" AND NOT (trial OR study))) OR (TITLE-ABS (nonrandom* AND NOT random*)) OR (TITLE-ABS ("random field*")) OR (TITLE-ABS ("random cluster" W/4 sampl*)) OR ((ABS (review) AND DOCTYPE (re)) AND NOT TITLE (trial)) OR (ABS ("we searched") AND DOCTYPE (re)) OR (ABS ("update review")) OR (ABS (databases W/5 searched)))))

CINAHL – RCTs

**CINAHL (EBSCOhost)**		
**#**	**Query**	**Results**
S50	S26 AND S49	2,351
S49	S48 NOT S47	924,141
S48	S27 OR S28 OR S29 OR S30 OR S31 OR S32 OR S33 OR S34 OR S35 OR S36 OR S37 OR S38 OR S39 OR S40 OR S41	969,980

S47	S45 NOT S46	207,909
S46	MH (human)	2,579,598
S45	S42 OR S43 OR S44	241,236
S44	TI (animal model*)	3,557
S43	MH (animal studies)	148,001
S42	MH animals+	102,031
S41	AB (cluster W3 RCT)	469
S40	MH (crossover design) OR MH (comparative studies)	459,276
S39	AB (control W5 group)	136,924
S38	PT (randomized controlled trial)	144,727
S37	MH (placebos)	13,408
S36	MH (sample size) AND AB (assigned OR allocated OR control)	4,364
S35	TI (trial)	168,327
S34	AB (random*)	381,042
S33	TI (randomised OR randomized)	130,724
S32	MH cluster sample	5,052
S31	MH pretest-posttest design	50,180
S30	MH random assignment	75,178

S29	MH single-blind studies	15,699
S28	MH double-blind studies	53,286
S27	MH randomized controlled trials	131,462
S26	S20 NOT S25	5,369
S25	S23 NOT S24	140,030
S24	TI nonpregnan* or non pregnan*	1,024
S23	S21 OR S22	140,915
S22	TI pregnan* or gestation* or primigravida? or multigravida? or secundigravida?	138,088
S21	(MM "Pregnancy") OR (MM "Expectant Mothers")	11,056
S20	S11 AND S19	5,411
S19	S12 OR S13 OR S14 OR S15 OR S16 OR S17 OR S18	68,210
S18	TI (maxim* oxygen or peak oxygen or VO2*) OR AB (maxim* oxygen or peak oxygen or VO2*)	11,327
S17	TI ((Submaximal or maximal or graded) N3 (treadmill? or tread mill? or ergometer?)) OR AB ((Submaximal or maximal or graded) N3 (treadmill? or tread mill? or ergometer?))	1,269
S16	TI ((functional or aerobic or exercise) N3 (capacity or endurance)) OR AB ((functional or aerobic or exercise) N3 (capacity or endurance))	16,140
S15	TI ((fitness or exercise or endurance or step or walk or run or beep or tread?mill or ergometry or eurofit or stress) N3 test*) OR AB ((fitness or	25,791

	exercise or endurance or step or walk or run or beep or tread?mill or ergometry or eurofit or stress) N3 test*)	
S14	TI ((minute or mile or distance or timed) N3 run) OR AB ((minute or mile or distance or timed) N3 run)	482
S13	TI ((cardiorespirator* or cardio respirator* or aerobic or cardiopulmonar* or cardio pulmonar* or cardiovascular* or cardio vascular* or physical* work* or cardiometaboli* or cardio metaboli*) N4 (fitness or capacit* or endurance or perform* or health*)) OR AB ((cardiorespirator* or cardio respirator* or aerobic or cardiopulmonar* or cardio pulmonar* or cardiovascular* or cardio vascular* or physical* work* or cardiometaboli* or cardio metaboli*) N4 (fitness or capacit* or endurance or perform* or health*))	25,524
S12	(MM "Cardiorespiratory Fitness") or (MM "Exercise Test+")	9,696
S11	S1 OR S2 OR S3 OR S4 OR S5 OR S6 OR S7 OR S8 OR S9 OR S10	90,220
S10	TI ((((video or computer or console) N1 game?) or videogam* or gaming)) OR AB ((((video or computer or console) N1 game?) or videogam* or gaming))	5,639
S9	TI ((brows* or stream* or surf* or time or use? or using or watch*) N3 (internet or web* or net)) OR AB ((brows* or stream* or surf* or time or use? or using or watch*) N3 (internet or web* or net))	16,311
S8	TI (((apple or samsung or android or amazon or google or computer? or time or watch* or view* or play* or stream* or game? or gaming) N3 tablet?) or ipad?)) OR AB (((apple or samsung or android or amazon or google or computer? or time or watch* or view* or play* or stream* or game? or gaming) N3 tablet?) or ipad?))	2,631
S7	TI ((television? or tv or "t.v.")) OR AB ((television? or tv or "t.v."))	12,783
S6	TI ((video? N2 (stream* or watch* or time or view*))) OR AB ((video? N2 (stream* or watch* or time or view*)))	3,396

S5	TI ((screen? N2 (time or watch* or view*))) OR AB ((screen? N2 (time or watch* or view*)))	2,668
S4	TI ((sitting or sit or sits or seated or recline? or reclining or lying or "lie down" or "lay down" or "laying down" or deskbound or (bound N1 (desk? or chair?)))) OR AB ((sitting or sit or sits or seated or recline? or reclining or lying or "lie down" or "lay down" or "laying down" or deskbound or (bound N1 (desk? or chair?))))	21,659
S3	TI (physical* N3 inactiv*) OR AB (physical* N3 inactiv*)	5,503
S2	TI (sedentar* or stationary or stationery) OR AB (sedentar* or stationary or stationery)	17,406
S1	(MM "Life style, sedentary") or (MM "sitting") or (MM "screen time") or (MM "video games" or (MM "television") or (MM "internet addiction") or (MM "Computers, Portable+")	20,587

CINAHL – Remaining Studies

**CINAHL (EBSCOhost)**		
**#**	**Query**	**Results**
S52	S51 NOT S48	2,889
S51	S26 NOT S50	3,022
S50	S26 AND S49	2,354
S49	S42 NOT S48	924,527
S48	S46 NOT S47	207,956

S47	MH (human)	2,580,538
S46	S43 OR S44 OR S45	241,284
S45	TI (animal model*)	3,558
S44	MH (animal studies)	148,060
S43	MH animals+	102,047
S42	S27 OR S28 OR S29 OR S30 OR S31 OR S32 OR S33 OR S34 OR S35 OR S36 OR S37 OR S38 OR S39 OR S40 OR S41	970,594
S41	AB (cluster W3 RCT)	469
S40	MH (crossover design) OR MH (comparative studies)	459,402
S39	AB (control W5 group)	137,005
S38	PT (randomized controlled trial)	144,790
S37	MH (placebos)	13,411
S36	MH (sample size) AND AB (assigned OR allocated OR control)	4,365
S35	TI (trial)	168,448
S34	AB (random*)	381,410
S33	TI (randomised OR randomized)	130,824
S32	MH cluster sample	5,052
S31	MH pretest-posttest design	50,212

S30	MH random assignment	75,218
S29	MH single-blind studies	15,701
S28	MH double-blind studies	53,293
S27	MH randomized controlled trials	131,527
S26	S20 NOT S25	5,376
S25	S23 NOT S24	140,122
S24	TI nonpregnan* or non pregnan*	1,024
S23	S21 OR S22	141,007
S22	TI pregnan* or gestation* or primigravida? or multigravida? or secundigravida?	138,175
S21	(MM "Pregnancy") OR (MM "Expectant Mothers")	11,070
S20	S11 AND S19	5,414
S19	S12 OR S13 OR S14 OR S15 OR S16 OR S17 OR S18	68,237
S18	TI (maxim* oxygen or peak oxygen or VO2*) OR AB (maxim* oxygen or peak oxygen or VO2*)	11,332
S17	TI ((Submaximal or maximal or graded) N3 (treadmill? or tread mill? or ergometer?)) OR AB ((Submaximal or maximal or graded) N3 (treadmill? or tread mill? or ergometer?))	1,269
S16	TI ((functional or aerobic or exercise) N3 (capacity or endurance)) OR AB ((functional or aerobic or exercise) N3 (capacity or endurance))	16,152

S15	TI ((fitness or exercise or endurance or step or walk or run or beep or tread?mill or ergometry or eurofit or stress) N3 test*) OR AB ((fitness or exercise or endurance or step or walk or run or beep or tread?mill or ergometry or eurofit or stress) N3 test*)	25,802
S14	TI ((minute or mile or distance or timed) N3 run) OR AB ((minute or mile or distance or timed) N3 run)	482
S13	TI ((cardiorespirator* or cardio respirator* or aerobic or cardiopulmonar* or cardio pulmonar* or cardiovascular* or cardio vascular* or physical* work* or cardiometaboli* or cardio metaboli*) N4 (fitness or capacit* or endurance or perform* or health*)) OR AB ((cardiorespirator* or cardio respirator* or aerobic or cardiopulmonar* or cardio pulmonar* or cardiovascular* or cardio vascular* or physical* work* or cardiometaboli* or cardio metaboli*) N4 (fitness or capacit* or endurance or perform* or health*))	25,538
S12	(MM "Cardiorespiratory Fitness") or (MM "Exercise Test+")	9,697
S11	S1 OR S2 OR S3 OR S4 OR S5 OR S6 OR S7 OR S8 OR S9 OR S10	90,299
S10	TI ((((video or computer or console) N1 game?) or videogam* or gaming)) OR AB ((((video or computer or console) N1 game?) or videogam* or gaming))	5,646
S9	TI ((brows* or stream* or surf* or time or use? or using or watch*) N3 (internet or web* or net)) OR AB ((brows* or stream* or surf* or time or use? or using or watch*) N3 (internet or web* or net))	16,319
S8	TI (((apple or samsung or android or amazon or google or computer? or time or watch* or view* or play* or stream* or game? or gaming) N3 tablet?) or ipad?)) OR AB (((apple or samsung or android or amazon or google or computer? or time or watch* or view* or play* or stream* or game? or gaming) N3 tablet?) or ipad?))	2,632
S7	TI ((television? or tv or "t.v.")) OR AB ((television? or tv or "t.v."))	12,786

S6	TI ((video? N2 (stream* or watch* or time or view*))) OR AB ((video? N2 (stream* or watch* or time or view*)))	3,397
S5	TI ((screen? N2 (time or watch* or view*))) OR AB ((screen? N2 (time or watch* or view*)))	2,670
S4	TI ((sitting or sit or sits or seated or recline? or reclining or lying or "lie down" or "lay down" or "laying down" or deskbound or (bound N1 (desk? or chair?)))) OR AB ((sitting or sit or sits or seated or recline? or reclining or lying or "lie down" or "lay down" or "laying down" or deskbound or (bound N1 (desk? or chair?))))	21,692
S3	TI (physical* N3 inactiv*) OR AB (physical* N3 inactiv*)	5,505
S2	TI (sedentar* or stationary or stationery) OR AB (sedentar* or stationary or stationery)	17,420
S1	(MM "Life style, sedentary") or (MM "sitting") or (MM "screen time") or (MM "video games" or (MM "television") or (MM "internet addiction") or (MM "Computers, Portable+")	20,591

SPORTDiscus - RCTs

**SPORTDiscus (EBSCOhost)**		
**#**	**Query**	**Results**
S36	S27 AND S35	1,114
S35	S28 OR S29 OR S30 OR S31 OR S32 OR S33 OR S34	83,266
S34	TI ((singl* or doubl* or tripl* or trebl*) N1 (blind* or dumm* or mask*)) OR AB ((singl* or doubl* or tripl* or trebl*) N1 (blind* or dumm* or mask*))	9,692
S33	TI (trial?) OR AB (trial?)	62,110
S32	TI (allocat* N3 (random or randomly)) OR AB (allocat* N3 (random or randomly))	1,959

S31	TI (randomi#ed) OR AB (randomi#ed)	37,748
S30	DE "CLINICAL trials"	10,183
S29	DE "BLIND experiment"	3,767
S28	DE "RANDOMIZED controlled trials"	16,318
S27	S21 NOT S26	5,521
S26	S24 NOT S25	7,654
S25	TI (nonpregnan* or non pregnan*)	83
S24	S22 OR S23	7,723
S23	TI (pregnan* or gestation* or primigravida? or multigravida? or secundigravida?)	5,029
S22	DE "PREGNANCY" OR DE "PREGNANT women"	5,390
S21	S11 AND S20	5,552
S20	S12 OR S13 OR S14 OR S15 OR S16 OR S17 OR S18 OR S19	59,253
S19	TI (maxim* oxygen or peak oxygen or VO2*) OR AB (maxim* oxygen or peak oxygen or VO2*)	18,248
S18	TI ((Submaximal or maximal or graded) N3 (treadmill? or tread mill? or ergometer?)) OR AB ((Submaximal or maximal or graded) N3 (treadmill? or tread mill? or ergometer?))	1,942
S17	TI ((functional or aerobic or exercise) N3 (capacity or endurance)) OR AB ((functional or aerobic or exercise) N3 (capacity or endurance))	12,593
S16	TI ((fitness or exercise or endurance or step or walk or run or beep or tread?mill or ergometry or eurofit or stress) N3 test*) OR AB ((fitness or exercise or endurance or step or walk or run or beep or tread?mill or ergometry or eurofit or stress) N3 test*)	19,786
S15	TI ((minute or mile or distance or timed) N3 run) OR AB ((minute or mile or distance or timed) N3 run) TI ((fitness or exercise or endurance or step or walk or run or beep or tread?mill or ergometry or eurofit or stress) N3 test*) OR AB ((fitness or exercise or endurance or step or walk or run or beep or tread?mill or ergometry or eurofit or stress) N3 test*)	17,505
S14	DE "EXERCISE tests" OR DE "STRESS echocardiography" OR DE "TREADMILL exercise tests"	11,087
S13	TI ((cardiorespirator* or cardio respirator* or aerobic or cardiopulmonar* or cardio pulmonar* or cardiovascular* or cardio vascular* or physical* work* or cardiometaboli* or cardio metaboli*) N4 (fitness or capacit* or	18,625

	endurance or perform* or health*)) OR AB ((cardiorespirator* or cardio respirator* or aerobic or cardiopulmonar* or cardio pulmonar* or cardiovascular* or cardio vascular* or physical* work* or cardiometaboli* or cardio metaboli*) N4 (fitness or capacit* or endurance or perform* or health*))	
S12	DE "CARDIOVASCULAR fitness" OR DE "CARDIOPULMONARY fitness"	2,024
S11	S1 OR S2 OR S3 OR S4 OR S5 OR S6 OR S7 OR S8 OR S9 OR S10	63,422
S10	TI ((((video or computer or console) N1 game?) or videogam* or gaming)) OR AB ((((video or computer or console) N1 game?) or videogam* or gaming))	4,146
S9	TI ((brows* or stream* or surf* or time or use? or using or watch*) N3 (internet or web* or net)) OR AB ((brows* or stream* or surf* or time or use? or using or watch*) N3 (internet or web* or net))	2,783
S8	TI (((apple or samsung or android or amazon or google or computer? or time or watch* or view* or play* or stream* or game? or gaming) N3 tablet?) or ipad?)) OR AB (((apple or samsung or android or amazon or google or computer? or time or watch* or view* or play* or stream* or game? or gaming) N3 tablet?) or ipad?))	606
S7	TI ((television? or tv or "t.v.")) OR AB ((television? or tv or "t.v."))	21,687
S6	TI ((video? N2 (stream* or watch* or time or view*))) OR AB ((video? N2 (stream* or watch* or time or view*)))	1,151
S5	TI ((screen? N2 (time or watch* or view*))) OR AB ((screen? N2 (time or watch* or view*)))	674
S4	TI ((sitting or sit or sits or seated or recline? or reclining or lying or "lie down" or "lay down" or "laying down" or deskbound or (bound N1 (desk? or chair?)))) OR AB ((sitting or sit or sits or seated or recline? or reclining or lying or "lie down" or "lay down" or "laying down" or deskbound or (bound N1 (desk? or chair?))))	15,152
S3	TI (physical* N3 inactiv*) OR AB (physical* N3 inactiv*)	2,489
S2	TI (sedentar* or stationary or stationery) OR AB (sedentar* or stationary or stationery)	13,113
S1	DE "SEDENTARY behavior" OR DE "SEDENTARY lifestyles" OR DE "SEDENTARY people" OR DE "SEDENTARY women" OR DE "SITTING position" OR DE "VIDEO games" OR DE "INTERNET"	11,487

SPORTDiscus – Remaining Studies

SPORTDiscus (EBSCOhost)		
#	Query	Results
S37	S27 NOT S36	4,413
S36	S27 AND S35	1,114
S35	S28 OR S29 OR S30 OR S31 OR S32 OR S33 OR S34	83,295
S34	TI ((singl* or doubl* or tripl* or trebl*) N1 (blind* or dumm* or mask*)) OR AB ((singl* or doubl* or tripl* or trebl*) N1 (blind* or dumm* or mask*))	9,695
S33	TI (trial?) OR AB (trial?)	62,128
S32	TI (allocat* N3 (random or randomly)) OR AB (allocat* N3 (random or randomly))	1,962
S31	TI (randomi#ed) OR AB (randomi#ed)	37,773
S30	DE "CLINICAL trials"	10,183
S29	DE "BLIND experiment"	3,767
S28	DE "RANDOMIZED controlled trials"	16,318
S27	S21 NOT S26	5,527
S26	S24 NOT S25	7,655
S25	TI (nonpregnan* or non pregnan*)	83
S24	S22 OR S23	7,724
S23	TI (pregnan* or gestation* or primigravida? or multigravida? or secundigravida?)	5,030
S22	DE "PREGNANCY" OR DE "PREGNANT women"	5,390
S21	S11 AND S20	5,558
S20	S12 OR S13 OR S14 OR S15 OR S16 OR S17 OR S18 OR S19	59,280
S19	TI (maxim* oxygen or peak oxygen or VO2*) OR AB (maxim* oxygen or peak oxygen or VO2*)	18,257
S18	TI ((Submaximal or maximal or graded) N3 (treadmill? or tread mill? or ergometer?)) OR AB ((Submaximal or maximal or graded) N3 (treadmill? or tread mill? or ergometer?))	1,942
S17	TI ((functional or aerobic or exercise) N3 (capacity or endurance)) OR AB ((functional or aerobic or exercise) N3 (capacity or endurance))	12,604

S16	TI ((fitness or exercise or endurance or step or walk or run or beep or tread?mill or ergometry or eurofit or stress) N3 test*) OR AB ((fitness or exercise or endurance or step or walk or run or beep or tread?mill or ergometry or eurofit or stress) N3 test*)	19,794
S15	TI ((minute or mile or distance or timed) N3 run) OR AB ((minute or mile or distance or timed) N3 run) TI ((fitness or exercise or endurance or step or walk or run or beep or tread?mill or ergometry or eurofit or stress) N3 test*) OR AB ((fitness or exercise or endurance or step or walk or run or beep or tread?mill or ergometry or eurofit or stress) N3 test*)	17,513
S14	DE "EXERCISE tests" OR DE "STRESS echocardiography" OR DE "TREADMILL exercise tests"	11,088
S13	TI ((cardiorespirator* or cardio respirator* or aerobic or cardiopulmonar* or cardio pulmonar* or cardiovascular* or cardio vascular* or physical* work* or cardiometaboli* or cardio metaboli*) N4 (fitness or capacit* or endurance or perform* or health*)) OR AB ((cardiorespirator* or cardio respirator* or aerobic or cardiopulmonar* or cardio pulmonar* or cardiovascular* or cardio vascular* or physical* work* or cardiometaboli* or cardio metaboli*) N4 (fitness or capacit* or endurance or perform* or health*))	18,635
S12	DE "CARDIOVASCULAR fitness" OR DE "CARDIOPULMONARY fitness"	2,025
S11	S1 OR S2 OR S3 OR S4 OR S5 OR S6 OR S7 OR S8 OR S9 OR S10	63,440
S10	TI ((((video or computer or console) N1 game?) or videogam* or gaming)) OR AB ((((video or computer or console) N1 game?) or videogam* or gaming))	4,146
S9	TI ((brows* or stream* or surf* or time or use? or using or watch*) N3 (internet or web* or net)) OR AB ((brows* or stream* or surf* or time or use? or using or watch*) N3 (internet or web* or net))	2,784
S8	TI (((apple or samsung or android or amazon or google or computer? or time or watch* or view* or play* or stream* or game? or gaming) N3 tablet?) or ipad?)) OR AB (((apple or samsung or android or amazon or google or computer? or time or watch* or view* or play* or stream* or game? or gaming) N3 tablet?) or ipad?))	606
S7	TI ((television? or tv or "t.v.")) OR AB ((television? or tv or "t.v."))	21,690
S6	TI ((video? N2 (stream* or watch* or time or view*))) OR AB ((video? N2 (stream* or watch* or time or view*)))	1,151
S5	TI ((screen? N2 (time or watch* or view*))) OR AB ((screen? N2 (time or watch* or view*)))	674
S4	TI ((sitting or sit or sits or seated or recline? or reclining or lying or "lie down" or "lay down" or "laying down" or deskbound or (bound N1 (desk?	15,159

	or chair?)))) OR AB ((sitting or sit or sits or seated or recline? or reclining or lying or "lie down" or "lay down" or "laying down" or deskbound or (bound N1 (desk? or chair?))))	
S3	TI (physical* N3 inactiv*) OR AB (physical* N3 inactiv*)	2,489
S2	TI (sedentar* or stationary or stationery) OR AB (sedentar* or stationary or stationery)	13,120
S1	DE "SEDENTARY behavior" OR DE "SEDENTARY lifestyles" OR DE "SEDENTARY people" OR DE "SEDENTARY women" OR DE "SITTING position" OR DE "VIDEO games" OR DE "INTERNET"	11,487
