# Supplementary material for: The Effect of Sedentary Behaviour on Cardiorespiratory Fitness: A Systematic Review and Meta-Analysis
Source: Sports Med. 2024 Jan 16;54(4):997–1013. doi: 10.1007/s40279-023-01986-y (PMC11052788; doi:10.1007/s40279-023-01986-y)
Supplement: Supplementary file 2 — Supplementary file2 (PDF 180 KB) [file 40279_2023_1986_MOESM2_ESM.pdf]

Title: The effect of sedentary behaviour on cardiorespiratory fitness: a systematic review and meta-analysis.
Journal: Sports Medicine
Authors: Stephanie A. Prince*, Paddy C. Dempsey, Jennifer L. Reed, Lukas Rubin, Travis J. Saunders, Josephine Ta, Grant R. Tomkinson, Katherine Merucci, Justin J. Lang
***Corresponding author:** Centre for Surveillance and Applied Research, Public Health Agency of Canada, stephanie.prince.ware@phac-aspc.gc.ca

Table S2. Summary of Decision Rules: GRADE Assessments for Certainty of Evidence from primary studies included per outcome

Domain	Judgment	Scoring	Criteria
**Risk of Bias (review quality)**	No serious ROB	0	All primary studies assessed as having a low risk of bias with no study limitations observed across studies.
	Serious, borderline ROB	–1 point	All primary studies were assessed to be at low to moderate risk of bias with a potential for bias arising from a lack of clarity on eligibility criteria, measurement of exposure or outcome, confounding, and/or loss to follow-up.
	Serious ROB	–2 points	• At least one of the primary studies was assessed to be of high risk of bias. The relative contribution of each study to the overall results will be considered for assessing the overall level of bias.<br>• Risk of biases include not having appropriate eligibility criteria and the populations are not generalizable, there is a serious flaw in the measurement of the exposure (non-exercise prediction equations vs. exercise-based measures) and/or outcome (i.e., self-report vs. medical records), there is a failure to adequately control for confounding (i.e., age, sex), and/or a loss to follow-up >10%. Biases related to generalizability, measurement of exposure and adequate control for confounding are considered the most important for the study.
**Inconsistency[a]**	No serious inconsistency	0	• There is minimal variance in the estimates across studies with overlap in the confidence intervals<br>• If meta-analysis is conducted, tests of heterogeneity are low to moderate and not significant.<br>• Heterogeneity can be explained by a priori established sub-group analyses.
	Serious inconsistency	–1 point	• There is observed variance in the estimates across studies, but most confidence intervals overlap<br>• If meta-analysis is conducted, tests of heterogeneity are at most moderate and significant.<br>• Heterogeneity could be partially (but not completely) explained by a priori established sub-group analyses.
	Very serious inconsistency	–2 points	• There is substantial variance in the estimates across studies, with minimal overlap in the confidence intervals<br>• If meta-analysis is conducted, tests of heterogeneity are significant and show substantial or considerable heterogeneity.<br>• Heterogeneity could not be explained by a priori established sub-group analyses.

**Indirectness**	No serious indirectness	0	• The included study PICOs are similar:<br>• Population characteristics (e.g., all general population, all desk-based workers, all individuals with same chronic condition)<br>• Interventions (i.e., focus on sedentary behaviour reduction or interrupting prolonged sitting, all single treatment or multicomponent with same alternate focus such as diet, physical activity, sleep)<br>• Comparator/control groups<br>• Outcome measures of CRF were consistent across studies
	Serious indirectness	–1 point	• There is some variation in the study population, intervention, comparator or CRF measures, but the variation was limited to only one PICO component<br>• Estimates were consistent regardless of differences in PICOs.
	Very serious indirectness	–2 points	• There is considerable variation in more than one of the PICO components: study population, intervention, comparator or CRF measures
**Imprecision**	No serious imprecision	0	• The estimated optimal information size (OIS) of a sample size of 134 per group was achieved.[a]<br>• The 95% CIs are narrow, or if they are wide, a very large sample size has been achieved (2000 per group).
	Serious imprecision	–1 point	The OIS of 134 participants per group is met, but the 95% CIs are wide.
	Very serious imprecision	–2 points	The OIS of 134 participants per group is not met.
**Publication Bias**	No serious risk of publication bias	0	There is no clear evidence of publication bias as assessed by a visual inspection of funnel plots or egger's test.<br><br>Publication bias will only be assessed if there are a minimum of 10 included studies. If there are fewer than 10 studies, no downgrade will occur.
	Serious risk of publication bias	-1 point	There is clear evidence of publication bias as assessed by a visual inspection of funnel plots or egger's test.

CRF = cardiorespiratory fitness, GRADE = Grading of Recommendations Assessment, Development and Evaluation, OIS = optimal information size, ROB = risk of bias

[a]OIS calculated using a sample size calculator (https://www.stat.ubc.ca/~rollin/stats/ssize/n2.html) with parameters: mean CRF value group 1 = 36.3 mL/kg/min based on women aged 20-29 years CPX data from cycle and treadmill tests (Kaminsky et al., 2022), group 2 = 39.8 mL/kg/min (representing a 1-MET or 3.5 mL/kg/min difference) (Kodama et al., 2009), sigma = 10.2 (Kaminsky et al., 2022), alpha = 0.05, desired power = 0.80, 2-sided test.

Notes: The quality of the evidence from RCT studies will initially be assigned as "high", whereas those relying on non-randomized designs will initially be assigned as "low".

- The quality of the evidence will be upgraded, if there is not cause to downgrade, and there is evidence of a large magnitude or dose-response gradient of effect from meta-analyses.
- The quality of the evidence will be interpreted as follows:
  High – we are confident that the true effect of sedentary behaviour on CRF is similar to the estimated effect and further research is very unlikely to change our confidence in the effect
  Moderate – we are moderately confident that the true effect of sedentary behaviour on CRF is likely to be close to the effect we have estimated, but there is a possibility that it is substantially different; further research is likely to have an important impact on the confidence in the effect
  Low – we have limited confidence; the true effect of sedentary behaviour on CRF may be substantially different from our estimate
  Very low – we have very little confidence; the true effect between sedentary behaviour and CRF is likely substantially different from our estimate
