# Supplementary material for: The Effect of Sedentary Behaviour on Cardiorespiratory Fitness: A Systematic Review and Meta-Analysis
Source: Sports Med. 2024 Jan 16;54(4):997–1013. doi: 10.1007/s40279-023-01986-y (PMC11052788; doi:10.1007/s40279-023-01986-y)
Supplement: Supplementary file 3 — Supplementary file3 (PDF 401 KB) [file 40279_2023_1986_MOESM3_ESM.pdf]

Title: The effect of sedentary behaviour on cardiorespiratory fitness: a systematic review and meta-analysis.
Journal: Sports Medicine
Authors: Stephanie A. Prince*, Paddy C. Dempsey, Jennifer L. Reed, Lukas Rubin, Travis J. Saunders, Josephine Ta, Grant R. Tomkinson, Katherine Merucci, Justin J. Lang
***Corresponding author:** Centre for Surveillance and Applied Research, Public Health Agency of Canada, stephanie.prince.ware@phac-aspc.gc.ca

Table S3. Excluded full texts and reasons for exclusion.

Study	Title	Journal	Exclusion reasons
**RCT filter full-text exclusions**			
Allan 2008	A tailored Internet intervention did not increase physical activity more than a tailored print intervention or publicly available web sites	Evidence Based Nurs	Abstract only
Andersen 2013	Cardiovascular health effects of internet-based encouragements to do daily workplace stair-walks: Randomized controlled trial	J Med Internet Res	No measure of SB
Atlantis 2006	Worksite intervention effects on physical health: a randomized controlled trial	Health Promot Int	No SB intervention component
Auerswald 2022	Impact of activity tracker usage in combination with a physical activity intervention on physical and cognitive parameters in healthy adults aged 60+: a randomized controlled trial	Int J Environ Res Public Health	No SB intervention component
Baillot 2016	Impacts of Supervised Exercise Training in Addition to Interdisciplinary Lifestyle Management in Subjects Awaiting Bariatric Surgery: a Randomized Controlled Study	Obes Surg	No SB intervention component
Balducci 2022	Relationships of changes in physical activity and sedentary behavior with changes in physical fitness and cardiometabolic risk profile in individuals with Type 2 diabetes: The Italian Diabetes and Exercise Study 2 (IDES_2)	Diabetes Care	Duplicate study
Balducci 2012	Changes in physical fitness predict improvements in modifiable cardiovascular risk factors independently of body weight loss in subjects with type 2 diabetes participating in the Italian Diabetes and Exercise Study (IDES)	Diabetes Care	No SB intervention component

Baruth 2010	Psychosocial mediators of physical activity and fitness changes in the activity counseling trial	Ann Behav Med	No SB intervention component
Bennett 2007	Motivational interviewing to increase physical activity in long-term cancer survivors: a randomized controlled trial	Nurs Res	No SB intervention component
Bhave 2016	Effectiveness of a 5-year school-based intervention programme to reduce adiposity and improve fitness and lifestyle in Indian children; the SYM-KEM study	Arch Dis Child	No SB intervention component
Biffi 2018	Ferrari Corporate Wellness Program: results of a pilot analysis and the "drag" impact in the workplace	High Blood Pressure Cardiovasc Prev	No SB intervention component
Bojsen-Moller 2022	The effect of two multi-component behavior change interventions on cognitive functions	BMC Public Health	CRF not used as the outcome
BowdenDavies 2018	Short-term decreased physical activity with increased sedentary behaviour causes metabolic derangements and altered body composition: effects in individuals with and without a first-degree relative with type 2 diabetes	Diabetologia	No SB intervention component
Butler 2018	Can reducing sitting time in the university setting improve the cardiometabolic health of college students?	Diab Metab Syndrome Obes	No measure of CRF
Canning 2003	A randomized controlled trial of the effects of intensive sit-to-stand training after recent traumatic brain injury on sit-to-stand performance	Clin Rehabil	No SB intervention component
Carels 2004	Reducing cardiovascular risk factors in postmenopausal women through a lifestyle change intervention	J Women Health	No SB intervention component
Chen 2020	Home-based physical activity and diet intervention to improve physical function in advanced liver disease: a randomized pilot trial	Digestive Diseases and Sciences	No SB intervention component
Clemes 2022	The effectiveness of the Structured Health Intervention for Truckers (SHIFT): a cluster randomised controlled trial (RCT)	BMC Medicine	No measure of CRF
Courneya 2016	Effects of a structured exercise program on physical activity and fitness in colon cancer survivors: one year feasibility results from the CHALLENGE trial	Cancer Epidemiology, Biomarkers & Prevention	No SB intervention component
Direito 2015	Apps for IMproving FITness and Increasing Physical Activity Among Young People: The AIMFIT pragmatic randomized controlled trial	J Med Internet Res	No SB intervention component

Dorling 2021	Effect of an office-based intervention on visceral adipose tissue: the WorkACTIVE-P randomized controlled trial	App Physiol Nutr Metab	No measure of CRF
Duck 2021	Physical activity and fitness: The feasibility and preliminary effectiveness of wearable activity tracker technology incorporating altruistic motivation in youth	Journal for Specialists in Pediatric Nursing	No SB intervention component
Dunn 1998	Six-month physical activity and fitness changes in Project Active, a randomized trial	Med Sci Sports Exerc	No SB intervention component
Dunn 1997	Reduction in cardiovascular disease risk factors: 6-month results from Project Active	Prev Med	No SB intervention component
Dunn 1999	Comparison of lifestyle and structured interventions to increase physical activity and cardiorespiratory fitness: a randomized trial	JAMA	No SB intervention component
Eather 2013	Improving the fitness and physical activity levels of primary school children: Results of the Fit-4-Fun group randomized controlled trial	Prev Med	No measure of SB
Ensenyat 2017	Metabolic risk management, physical exercise and lifestyle counselling in low-active adults: controlled randomized trial	BMC Public Health	Study protocol
Godoy-Izquierdo 2017	Improvements in health-related quality of life, cardio-metabolic health, and fitness in postmenopausal women after a supervised, multicomponent, adapted exercise program in a suited health promotion intervention: a multigroup study	Menopause	No SB intervention component
Graf 2011	The CHILT I project (Children's Health Interventional Trial): A multicomponent intervention to prevent physical inactivity and overweight in primary schools	Bundesgesundheitsblatt Gesundheitsforschung Gesundheitsschutz	Not English, French, Spanish or Czech
Guinan 2017	Effects of a multimodal rehabilitation programme on inflammation and oxidative stress in oesophageal cancer survivors: the ReStOre feasibility study	Supportive Care in Cancer	No SB intervention component
Hageman 2005	Tailored versus standard Internet-delivered interventions to promote physical activity in older women	J Geriatr Phys Ther	No SB intervention component
Hansen 2012	Effect of a Web-based intervention to promote physical activity and improve health among physically inactive adults: a population-based randomized controlled trial	J Med Internet Res	No SB intervention component

Harrell 1996	An occupation based physical activity intervention program: improving fitness and decreasing obesity	AAOHN Journal	No SB intervention component
Healy 2017	A Cluster RCT to Reduce Workers' Sitting Time: Impact on Cardiometabolic Biomarkers	Med Sci Sports Exerc	No measure of CRF
Hopstock 2021	Changes in adiposity, physical activity, cardiometabolic risk factors, diet, physical capacity and well-being in inactive women and men aged 57-74 years with obesity and cardiovascular risk - A 6-month complex lifestyle intervention with 6-month follow-up	PLoS ONE	No SB intervention component
Huber 2022	Sustainability of hiking in combination with coaching in cardiorespiratory fitness and quality of life	Int J Environ Res Public Health	No SB intervention component
Ijzelenberg 2012	The effect of a comprehensive lifestyle intervention on cardiovascular risk factors in pharmacologically treated patients with stable cardiovascular disease compared to usual care: a randomised controlled trial	BMC Cardiovasc Disorders	No SB intervention component
Jackson 2022	A randomized clinical trial demonstrating feasibility and preliminary efficacy of a videoconference-delivered physical activity lifestyle intervention among adolescents with a congenital heart defect	Ann Behav Med	No SB intervention component
Jamner 2004	A controlled evaluation of a school-based intervention to promote physical activity among sedentary adolescent females: Project FAB	J Adoles Health	No SB intervention component
Kahana 2022	The effect of incorporating an exergame application in a multidisciplinary weight management program on physical activity and fitness indices in children with overweight and obesity	Children	No SB intervention component
Ligibel 2012	Impact of a telephone-based physical activity intervention upon exercise behaviors and fitness in cancer survivors enrolled in a cooperative group setting	Breast Cancer Res Treat	No SB intervention component
Little 2004	A randomised controlled trial of three pragmatic approaches to initiate increased physical activity in sedentary patients with risk factors for cardiovascular disease	Br J Gen Pract	No SB intervention component

Lonsdale 2021	Effect of a scalable school-based intervention on cardiorespiratory fitness in children: a cluster randomized clinical trial	JAMA Ped	No measure of SB
Lunde 2020	Long-term follow-up with a smartphone application improves exercise capacity post cardiac rehabilitation: A randomized controlled trial	Eur J Prev Cardiol	No SB intervention component
Mailey 2022	Reducing occupational sitting while working from home: individual and combined effects of a height-adjustable desk and an online behavioral intervention	J Occup Environ Med	No measure of CRF
Marcus 2007	Step into Motion: A randomized trial examining the relative efficacy of Internet vs. print-based physical activity interventions	Contemporary Clinical Trials	Study protocol
Marechal 2019	Effect of a mixed-exercise program on physical capacity and sedentary behavior in older adults during cancer treatments	Aging Clinical Exper Res	No SB intervention component
Mascaro 2022	effect of a six-month lifestyle intervention on the physical activity and fitness status of adults with NAFLD and metabolic syndrome	Nutrients	No SB intervention component
Mascola 2009	Framing physical activity as a distinct and uniquely valuable behavior independent of weight management: A pilot randomized controlled trial for overweight and obese sedentary persons	Eating Weight Disorders	No SB intervention component
Maylor 2018	Efficacy of a multicomponent intervention to reduce workplace sitting time in office workers a cluster randomized controlled trial	J Occup Environ Med	No measure of CRF
McGrane 2018	Outcomes of the Y-PATH Randomized Controlled Trial: can a school-based intervention improve fundamental movement skill proficiency in adolescent youth?	J Phys Act Health	No SB intervention component
Melero-Canas 2020	Effects of an Educational Hybrid Physical Education Program on Physical Fitness, Body Composition and Sedentary and Physical Activity Times in Adolescents: The Seneb's Enigma	Frontiers Psychol	No SB intervention component
Mora-Gonzalez 2020	The "$in TIME" gamification project: using a mobile app to improve cardiorespiratory fitness levels of college students	Games Health J	No SB intervention component

Morowatisharifabad 2021	The effect of integrated intervention based on protection motivation theory and implementation intention to promote physical activity and physiological indicators of patients with Type 2 diabetes	BioMed Res Int	No SB intervention component
Obling 2013	The MILE study: a motivational, individual and locally anchored exercise intervention among 30-49 year-olds with low levels of cardiorespiratory fitness: a randomised controlled study in primary care	BMC Public Health	Study protocol
Ortiz 2021	Effectiveness of a home-based exercise intervention in the fitness profile of Hispanic survivors of breast cancer	Rehabil Oncology	No SB intervention component
Pedersen 2014	An e-health intervention designed to increase workday energy expenditure by reducing prolonged occupational sitting habits	Work	No measure of CRF
Petersen 2012	A population-based randomized controlled trial of the effect of combining a pedometer with an intervention toolkit on physical activity among individuals with low levels of physical activity or fitness	Prev Med	No SB intervention component
Plotnikoff 2017	Integrating smartphone technology, social support and the outdoor physical environment to improve fitness among adults at risk of, or diagnosed with, Type 2 Diabetes: Findings from the 'eCoFit' randomized controlled trial	Prev Med	No SB intervention component
Pullyblank 2020	Effects of the Strong Hearts, Healthy Communities Intervention on functional fitness of rural women	J Rural Health	No SB intervention component
Ramadi 2016	Long-term physical activity behavior after completion of traditional versus fast-track cardiac rehabilitation	J Cardiovasc Nurs	No SB intervention component
Rasmussen 2022	Comprehensive cardiac rehabilitation for patients following infective endocarditis: results of the randomized CopenHeartIEtrial	Eur J Cardiovasc Nurs	No SB intervention component
Rhodes 2021	Couple-based physical activity planning for new parents: a randomized trial	Ame J Prev Med	No SB intervention component
Rodriguez-Hernandez 2019	The effect of 2 walking programs on aerobic fitness, body composition, and physical activity in sedentary office employees	PLoS ONE	No SB intervention component

Rogers 2009	A randomized trial to increase physical activity in breast cancer survivors	Med Sci Sports Exerc	No SB intervention component
Roig-Coll 2020	Effects of Aerobic Exercise, Cognitive and Combined Training on Cognition in Physically Inactive Healthy Late-Middle-Aged Adults: The Projecte Moviment Randomized Controlled Trial	Frontiers Aging Neurosci	No SB intervention component
Romé 2009	Physical activity on prescription (PAP): costs and consequences of a randomized, controlled trial in primary healthcare	Scand J Primary Health Care	No SB intervention component
Seghers 2014	The added value of a brief self-efficacy coaching on the effectiveness of a 12-week physical activity program	J Phys Act Health	No SB intervention component
Sevick 2000	Cost-effectiveness of lifestyle and structured exercise interventions in sedentary adults. Results of project ACTIVE	Am J Prev Med	No SB intervention component
Slootmaker 2009	Feasibility and effectiveness of online physical activity advice based on a personal activity monitor: randomized controlled trial	J Med Internet Res	No SB intervention component
Slootmaker 2010	Accelerometers and Internet for physical activity promotion in youth? Feasibility and effectiveness of a minimal intervention	Prev Med	No measure of SB
Smolander 2000	Work ability, physical activity, and cardiorespiratory fitness: 2-year results from Project Active	J Occup Environ Med	No SB intervention component
Swift 2021	The Effect of Aerobic Training and Increasing Nonexercise Physical Activity on Cardiometabolic Risk Factors	Med Sci Sports Exerc	No measure of SB
Toraman 2005	Effects of six weeks of detraining on retention of functional fitness of old people after nine weeks of multicomponent training	Br J Sports Med	No SB intervention component
Tully 2007	Randomised controlled trial of home-based walking programmes at and below current recommended levels of exercise in sedentary adults	J Epi Comm Health	No measure of SB
VanRoie 2010	Effectiveness of a lifestyle physical activity versus a structured exercise intervention in older adults	J Aging Phys Act	No measure of SB
Vetter 2018	Learning "Math on the Move": effectiveness of a combined numeracy and physical activity program for primary school children	J Phys Act Health	No SB intervention component

Wilbur 2008	Outcomes of a home-based walking program for African-American women	Am J Health Promot	No SB intervention component
Wilbur 2016	Randomized Clinical Trial of the Women's Lifestyle Physical Activity Program for African-American Women: 24- and 48-Week Outcomes	Am J Health Promot	No SB intervention component
Woodward 2022	Supervised aerobic exercise training and increased lifestyle physical activity to reduce cardiovascular disease risk for women with polycystic ovary syndrome: a randomized controlled feasibility trial	J Phys Act Health	Study protocol
Wyke 2019	The effect of a programme to improve men's sedentary time and physical activity: The European fans in training (EuroFIT) randomised controlled trial	PLoS Med	No measure of CRF
Young 2006	Effects of a life skills intervention for increasing physical activity in adolescent girls	Arch Ped Adoles Med	No SB intervention component
Zongpa 2020	Effectiveness of a smartphone directed physical activity program on cardiometabolic disease risk in desk-based office employees -- a pragmatic, two-arm, parallel, cluster randomised trial	Muscles Ligaments Tendons J	No measure of SB
**RCT filter full-text exclusions**			
Marti 1989	Association of physical activity with coronary risk factors and physical ability: twenty-year follow-up of a cohort of Finnish men	Age Ageing	No SB exposure/intervention
Cohen 1991	Long-term effects of a lifestyle modification exercise program on the fitness of sedentary, obese children	J Sports Med Phys Fit	No SB exposure/intervention
Janz 2000	Tracking physical fitness and physical activity from childhood to adolescence: the Muscatine study	Med Sci Sports Exerc	Did not assess change in SB and change in CRF
Ransdell 2003	Daughters and mothers exercising together: effects of home- and community-based programs	Med Sci Sports Exerc	No SB exposure/intervention
Fincher 2004	Tailored interventions to increase physical activity and cardiorespiratory fitness in younger black females		Thesis
Ohta 2004	The effect of lifestyle modification on physical fitness and work ability in different workstyles	J UOEH	No SB exposure/intervention
Toraman 2004	Age responses to multicomponent training programme in older adults	Disabil Rehabil	No SB exposure/intervention

Treuth 2004	A longitudinal study of fitness and activity in girls predisposed to obesity	Med Sci Sports Exerc	Did not assess change in SB and change in CRF
Photiou 2008	Lifestyle, Body Composition, and Physical Fitness Changes in Hungarian School Boys (1975-2005)	Res Quar Exerc Sport	Cross-sectional
Aires 2010	A 3-year longitudinal analysis of changes in fitness, physical activity, fatness and screen time	Acta paediatrica	Did not assess change in SB and change in CRF
Aires 2010	A 3-year longitudinal analysis of changes in body mass index	Int J Sports Med	Did not assess change in SB and change in CRF
Hutchens 2010	The impact of a pilot community intervention on health-related fitness measures in overweight children	Int J Exerc Sci	No SB exposure/intervention
Orlowski 2010	Physical activity in an organized after school program: a pilot study	J Exerc Physiol Online	No SB exposure/intervention
Sola 2010	An activity-based intervention for obese and physically inactive children organized in primary care: feasibility and impact on fitness and BMI: a one-year follow-up study	Scand J Primary Health Care	No SB exposure/intervention
VanRoie 2010	Effectiveness of a lifestyle physical activity versus a structured exercise intervention in older adults	J Aging Phys Act	No SB exposure/intervention
Baruth 2011	Changes in Physical Functioning in the Active Living Every Day Program of the Active for Life Initiative®	Int J Behav Med	No SB exposure/intervention
Farris 2011	A 12-week interdisciplinary intervention program for children who are obese	Cardiopulm Phys Ther J	No SB exposure/intervention
Machado-Rodrigues 2012	Cardiorespiratory fitness, weight status and objectively measured sedentary behaviour and physical activity in rural and urban Portuguese adolescents	J Child Health Care	Cross-sectional
Mota 2012	Cardiorespiratory fitness and TV viewing in relation with metabolic risk factors in youth	J Sci Med Sport	Conference abstract
Naidoo 2012	The impact of a primary school physical activity intervention in KwaZulu-Natal, South Africa	Afr J Phys Health Educ Rec Dance	No SB exposure/intervention
Saunders 2012	Effects of prolonged sitting and physical activity on markers of cardiometabolic risk in healthy children and youth: A pilot study	J Sci Med Sport	acute study
Shaibi 2012	Effects of a culturally grounded community-based diabetes prevention program for obese Latino adolescents	Diabetes Educator	No SB exposure/intervention

Toto 2012	Outcomes of a multicomponent physical activity program for sedentary, community-dwelling older adults	J Aging Phys Act	No SB exposure/intervention
Hjorth 2013	Seasonal variation in objectively measured physical activity, sedentary time, cardio-respiratory fitness and sleep duration among 8-11 year-old Danish children: a repeated-measures study	BMC Public Health	Did not assess change in SB and change in CRF
Ramli 2013	Effects of a worksite health programme on the improvement of physical health among overweight and obese civil servants: a pilot study	Malaysian J Med Sci	No SB exposure/intervention
Castillo-Rodriguez 2014	Cardiovascular program to improve physical fitness in those over 60 years old - pilot study	Clin Interventions Aging	No SB exposure/intervention
Cliff 2014	Volumes and bouts of sedentary behavior and physical activity: associations with cardiometabolic health in obese children	Obesity	No CRF outcome
Gomes 2014	Effects of a group physical activity program on physical fitness and quality of life in individuals with schizophrenia	Mental Health Phys Act	No SB exposure/intervention
Janssens 2014	An inactive lifestyle and low physical fitness are associated with functional somatic symptoms in adolescents. The TRAILS study	J Psychosomatic Res	Cross-sectional
Kelishadi 2014	Short-term effects of a physical activity intervention on obesity and aerobic fitness of adolescent girls	Int J Prev Med	No SB exposure/intervention
Kozey-Keadle 2014	Changes in Sedentary Time and Physical Activity in Response to an Exercise Training and/or Lifestyle Intervention	J Phys Act Health	No CRF outcome
Mantoani 2014	Long-term effects of a program to increase physical activity in smokers	Chest	No SB exposure/intervention
Sanders 2014	Effects of the Get Youth Moving (GYM) Intervention on Health-Related Fitness and Behaviors	J Park Rec Admin	No SB exposure/intervention
Shuval 2014	Sedentary behavior, cardiorespiratory fitness, physical activity, and cardiometabolic risk in men: the cooper center longitudinal study	Mayo Clinic Proceedings	Did not assess change in SB and change in CRF
Carter 2015	Energy expenditure and heart rate response to breaking up sedentary time with three different physical activity interventions	Nutr Metab Cardiovasc Dis	acute study

Greer 2015	The effects of sedentary behavior on metabolic syndrome independent of physical activity and cardiorespiratory fitness	J Phys Act Health	Did not assess change in SB and change in CRF
Hetherington 2015	Assessing the real world effectiveness of the Healthy Eating Activity and Lifestyle (HEAL) program	Health Promot J Australia	No SB exposure/intervention
Sanders 2015	Promoting Healthy Lifestyles to Children at School: Using a Multidisciplinary Train-the-Trainer Approach	J Public Health Manage Pract	No SB exposure/intervention
Uijtdewilligen 2015	Person-related determinants of TV viewing and computer time in a cohort of young Dutch adults: Who sits the most?	Scand J Med Sci Sports	Exclusion reason: No SB exposure/intervention
Caldwell 2016	Tracking of physical activity and fitness during the early years	Appl Physiol Nutr Metab	Did not assess change in SB and change in CRF
Lamb 2016	Prospective associations between sedentary time, physical activity, fitness and cardiometabolic risk factors in people with type 2 diabetes	Diabetologia	Did not assess change in SB and change in CRF
Ojala 2016	Outpatient rehabilitation as an intervention to improve employees' physical capacity	Work	No SB exposure/intervention
Cho 2017	Relationship Between Adherence to Individual Goals Within the 5-2-1-0 Guidelines for Obesity Prevention and Number of PACER Laps in Adolescents	J School Health	Did not assess change in SB and change in CRF
Da Cuna Carrera 2017	Sedentarism impact on cardiorespiratory response in college students	Revista Internacional de Medicina y Ciencias de la Actividad Fisica y del Deporte	Cross-sectional
Godoy-Izquierdo 2017	Improvements in health-related quality of life, cardio-metabolic health, and fitness in postmenopausal women after a supervised, multicomponent, adapted exercise program in a suited health promotion intervention: A multigroup study	Menopause	No SB exposure/intervention
Horswill 2017	Effect of a novel workstation device on promoting non-exercise activity thermogenesis (NEAT)	Work	acute study
Le 2017	A home-based physical activity intervention using activity trackers in survivors of childhood cancer: A pilot study	Ped Blood Cancer	No SB exposure/intervention

Leppanen 2017	Longitudinal physical activity, body composition, and physical fitness in preschoolers	Med Sci Sports Exerc	Duplicate from other search
Lin 2017	Short-Term Efficacy of a "Sit Less, Walk More" Workplace Intervention on Improving Cardiometabolic Health and Work Productivity in Office Workers	J Occup Environ Med	No CRF outcome
Mesquita 2017	Changes in physical activity and sedentary behaviour following pulmonary rehabilitation in patients with COPD	Resp Med	No SB exposure/intervention
Messiah 2017	Impact of a park-based afterschool program replicated over five years on modifiable cardiovascular disease risk factors	Prev Med	No SB exposure/intervention
BowdenDavies 2018	Short-term decreased physical activity with increased sedentary behaviour causes metabolic derangements and altered body composition: effects in individuals with and without a first-degree relative with type 2 diabetes	Diabetologia	No SB exposure/intervention
Butler 2018	Can reducing sitting time in the university setting improve the cardiometabolic health of college students?	Diabetes, metabolic syndrome Obes	No CRF outcome
Freene 2018	Objectively measured changes in physical activity and sedentary behavior in cardiac rehabilitation: a prospective cohort study	J Cardiopulm Rehab Prev	No SB exposure/intervention
Genin 2018	Effect of work-related sedentary time on overall health profile in active vs. inactive office workers	Frontiers Public Health	No SB exposure/intervention
O'Brien 2018	Achieving Canadian physical activity guidelines is associated with better vascular function independent of aerobic fitness and sedentary time in older adults	Appl Physiol Nutr Metab	Did not assess change in SB and change in CRF
Skrede 2018	Does cardiorespiratory fitness moderate the prospective association between physical activity and cardiometabolic risk factors in children?	Int J Obes	Did not assess change in SB and change in CRF
Gonsalves 2019	Clusters of health risk factors associated with cardiorespiratory fitness among adolescents	Eur J Prev Cardiol	Did not assess change in SB and change in CRF
Lopez-Torres 2019	Does fitness attenuate the relationship between changes in sitting time and health-related quality of life over time in community-dwelling older adults? Evidence from the EXERNET multicenter longitudinal study	Qual Life Res	Did not assess change in SB and change in CRF
Peterman 2019	Cardiometabolic effects of a workplace cycling intervention	J Phys Act Health	Duplicate from other search

Proudfoot 2019	Physical activity and trajectories of cardiovascular health indicators during early childhood	Pediatrics	No SB exposure/intervention
Ye 2019	Effects of school-based exergaming on urban children's physical activity and cardiorespiratory fitness: A quasi-experimental study	Int J Environ Res Public Health	No SB exposure/intervention
Blom 2020	Healthy Life Centres: A 3-month behaviour change programme's impact on participants' physical activity levels, aerobic fitness and obesity: An observational study	BMJ Open	No SB exposure/intervention
Camiletti-Moiron 2020	Changes in and the mediating role of physical activity in relation to active school transport, fitness and adiposity among Spanish youth: the UP&DOWN longitudinal study	Int J Behav Nutr Phys Act	No SB exposure/intervention
Harber 2020	Trends in cardiorespiratory fitness among apparently healthy adults from the Ball State Adult Fitness Longitudinal Lifestyle STudy (BALL ST) cohort from 1970-2019	PLoS ONE	No SB exposure/intervention
Kerroum 2020	Types of Classroom Chair Stretch Exercises and their Feasibility and Potential to Structure the Recommend Classroom-Based Physical Activity Break Primary Programs	Acta Facultatis Educationis Physicae Universitatis Comenianae	No SB exposure/intervention
Lopez-Roman 2020	Feasibility of implementing a preventive physical exercise programme recommended by general practitioners in cardiovascular risk patients: A pre-post comparison study	Eur J Gen Pract	No SB exposure/intervention
Mora-Gonzalez 2020	The "$in TIME" Gamification Project: Using a Mobile App to Improve Cardiorespiratory Fitness Levels of College Students	Games Health J	No SB exposure/intervention
Mossavar-Rahmani 2020	Are sedentary behavior and physical activity independently associated with cardiometabolic benefits? The Hispanic Community Health Study/Study of Latinos	BMC Public Health	No CRF outcome
Pippi 2020	Evaluation of maximum oxygen consumption in obese adolescents before and after a multidisciplinary lifestyle intervention	Gazzetta Medica Italiana Archivio per le Scienze Mediche	Not English, French, Spanish, Czech
Rosenbaum 2020	Changing health workforce attitudes to promote improved physical health in mental health service users: Keeping our Staff in Mind (KoSiM)	Health Promot J Aust	No SB exposure/intervention

Ulvestad 2020	Cardiorespiratory Fitness and Physical Activity following Lung Transplantation: A National Cohort Study	Respiration	No SB exposure/intervention
Whipple 2020	Influence of changes in sedentary time on outcomes of supervised exercise therapy in individuals with comorbid peripheral artery disease and Type 2 diabetes	Annals Vasc Surg	No SB exposure/intervention
Aguinaga 2021	BAILAMOS With mHealth Technology! Improving physical activity and well-being in middle-aged and older Latinxs: A pre-post feasibility study	Health Educ Behav	Duplicate from other search
Becker 2021	Run fast and sit still: Connections among aerobic fitness, physical activity, and sedentary time with executive function during pre-kindergarten	Early Child Res Quarterly	Did not assess change in SB and change in CRF
Beltran-Valls 2021	Longitudinal Associations of Healthy Behaviors on Fitness in Adolescents: DADOS Study	Am J Prev Med	Duplicate from other search
Graham 2021	Examining the acute effects of classroom-based physical activity breaks on executive functioning in 11- to 14-year-old children: single and additive moderation effects of physical fitness	Frontiers Ped	acute study
Griffiths 2021	Co-production at work: the process of breaking up sitting time to improve cardiovascular health. a pilot study	Int J Environ Res Public Health	No CRF outcome
Hopstock 2021	Changes in adiposity, physical activity, cardiometabolic risk factors, diet, physical capacity and well-being in inactive women and men aged 57-74 years with obesity and cardiovascular risk: A 6-month complex lifestyle intervention with 6-month follow-up	PLoS ONE	No SB exposure/intervention
Korhonen 2021	Longitudinal associations of physical activity, sedentary time, and cardiorespiratory fitness with arterial health in children the PANIC study	J Sports Sc	Did not assess change in SB and change in CRF
Larsen 2021	A multi-component intervention to affect physical activity, sleep length and stress levels in office workers	Smart Health	No SB exposure/intervention
Lona 2021	Changes in physical activity behavior and development of cardiovascular risk in children	Scand J Med Sci Sports	Did not assess change in SB and change in CRF
Master 2021	Joint Association of Moderate-to-vigorous Intensity Physical Activity and Sedentary Behavior with Incident Functional Limitation: Data from the Osteoarthritis Initiative	J Rheumatology	No CRF outcome

Morrison 2021	The effect of pandemic movement restriction policies on children's physical fitness, activity, screen time, and sleep	Frontiers Public Health	No SB exposure/intervention
O'Brien 2021	Examining the impact of a community-based exercise intervention on cardiorespiratory fitness, cardiovascular health, strength, flexibility and physical activity among adults living with HIV: A three-phased intervention study	PLoS ONE	No SB exposure/intervention
Reisberg 2021	Physical activity, fitness, and cognitive performance of estonian first-grade schoolchildren according their MVPA level in kindergarten: a longitudinal study	Int J Environ Res Public Health	Did not assess change in SB and change in CRF
Soh 2021	The effect of home-based intervention program on body composition, vo2 max and vital capacity among sedentary females leaving in unstable country	J Cardiovasc Dis Res	No SB exposure/intervention
Song 2021	Effects of school-based exercise program on obesity and physical fitness of urban youth: a quasi-experiment	Healthcare	No SB exposure/intervention
Ybarra 2021	Promoting healthy lifestyle behaviours in youth: Findings from a novel intervention for children at risk of cardiovascular disease	Paed Child Health	No SB exposure/intervention
Zabatiero 2021	Patterns of change in device-based physical activity and sedentary time following bariatric surgery: a longitudinal observational study	Obes Surg	Did not assess change in SB and change in CRF
AriasLabrador 2022	Efectos de un programa interdisciplinar combinado con entrenamiento aerbico continuo variable y fuerza dinÃ¡mica en el sÃndrome coronario agudo	Rehabilitacion	No SB exposure/intervention
Dougherty 2022	Five-year changes in objectively measured cardiorespiratory fitness, physical activity, and sedentary time in mid-to-late adulthood	Appl Physiol Nutr Metab	Did not assess change in SB and change in CRF
Guirado 2022	A 12-week cycling workstation intervention improves cardiometabolic risk factors in healthy inactive office workers	J Occup Environ Med	Duplicate from other search
Migueles 2022	Revisiting the cross-sectional and prospective association of physical activity with body composition and physical fitness in preschoolers: A compositional data approach	Ped Obes	Did not assess change in SB and change in CRF

Nebot 2022	Changes in physical fitness and body fat percentage: effects of a program focusing on health habits in children from 10 to 12 years old	J Sport Health Res	No SB exposure/intervention
