# Supplementary material for: The Effect of Sedentary Behaviour on Cardiorespiratory Fitness: A Systematic Review and Meta-Analysis
Source: Sports Med. 2024 Jan 16;54(4):997–1013. doi: 10.1007/s40279-023-01986-y (PMC11052788; doi:10.1007/s40279-023-01986-y)
Supplement: Supplementary file 4 — Supplementary file4 (PDF 303 KB) [file 40279_2023_1986_MOESM4_ESM.pdf]

Title: The effect of sedentary behaviour on cardiorespiratory fitness: a systematic review and meta-analysis.
Journal: Sports Medicine
Authors: Stephanie A. Prince*, Paddy C. Dempsey, Jennifer L. Reed, Lukas Rubin, Travis J. Saunders, Josephine Ta, Grant R. Tomkinson, Katherine Merucci, Justin J. Lang
***Corresponding author:** Centre for Surveillance and Applied Research, Public Health Agency of Canada, stephanie.prince.ware@phac-aspc.gc.ca

Table S4. Randomized controlled study characteristics

First author, year	Country	Population description	Age (SD)	Sample size analyzed				I length	SB target	Intervention strategy	PA intervention target	CRF outcome (test methods)
				I			C					
**Children and youth <18 years**												
Peralta, 2009	Australia	Secondary school children	12.5 (0.4)	16			17	6 months	Reduce screen time	Education, self-monitoring	1 x 60-min curriculum session + 2 x 20-min lunch time PA sessions/wk	Laps (maximal)
Robinson, 1999	USA	Elementary school students (3rd/4th grade)	I: 8.95 (0.6), C: 8.92 (0.7)	92			100	6 months	Reduce leisure screen time	Education, TV allowance device	None	Laps (maximal)
Sacher, 2010	UK	Children with obesity	I: 10.3 (1.3), C: 10.2 (1.3)	60			56	21 weeks	Reduce total SB	Education	2 x 1-h/wk	Recovery HR (submaximal)
Zhou, 2019	China	7th grade students	12.7 (0.6)	162 (SPE)	180 (ASP)	168 (SPE+ASP)	170	8 months	Reduce total and school	Education, environmental	Additional school-based MVPA	Laps (maximal)
**Adults ≥ 18 years**												
Aguinaga, 2019	USA	Older Latinos attending an adult wellness centre	75.4 (6.3)	10			11	4 months	Reduce, interrupt	Education, self-monitoring	2 h/week of dance	METS (regression equation, predicted)
Balducci, 2019	Italy	Physically inactive and sedentary patients with Type 2 diabetes	61.6 (8.5)	150			150	3 years	Reduce, interrupt	Education	8 x (30 min aerobic + 30 min resistance + 15 min warm up/ cool down)	$\dot{V}O_2$max (maximal, predicted)
Bergman, 2018	Sweden	Office workers with mainly sedentary tasks, middle-aged with overweight/obesity	I: 52.4 (6.8)	34			37	50 weeks	Reduce	Education, environmental	Asked to walk at least 1h/day on	Resting HR (n/a)

			C: 50.3 (6.7)								treadmill workstation	
Carr, 2013	USA	Sedentary, overweight university employees	44.7 (9.6)	23			17	12 weeks	Reduce	Environmental, prompts	None	$\dot{V}O_2$max (submaximal, predicted)
Carr, 2016	USA	Healthy, but physically inactive, overweight/obese adults working in full-time sedentary jobs (self-reported sitting >75% of workday)	I: 45.2 (10.9), C: 45.0 (10.7)	27			27	16 weeks	Reduce occupational ST	Educational, environmental, prompts, self-monitoring	Pedal ergometer, progressed from 30 min/day at Week 1 to 80 min/day	Resting HR (n/a)
Carter, 2020	UK	Healthy, university office workers	42.5 (10)	14			14	8 weeks	Reduce work, interrupt	Education, prompts	2 min walking breaks	Resting HR (n/a)
Cheng, 2022	Australia	COPD patients	I: 74 (9) C: 73 (10)	14			29	6 weeks	Reduce, interrupt	Education	None	6MWT distance (submaximal)
Dunning, 2018	South Africa	Desk based workers	27.5 (5.7)	11			9	10 weeks	Interrupt	Prompts	None	$\dot{V}O_2$max (maximal, direct)
Guirado, 2022	France	Healthy, sedentary and inactive workers	43.9 (8.7)	17			15	12 weeks	Reduce work	Environmental, self-monitoring	60 min/day	Resting HR (submaximal)
Kozey-Keadle, 2014	USA	Sedentary workers with overweight/ obesity at risk for cardiovascular disease	43.6	16 (EX-rST)	14 (rST)	16 (EX)	8	12 weeks	Reduce total and work	Education, self-monitoring	40 min x 5/wk in exercise groups	$\dot{V}O_2$peak (maximal, direct)
Larisch, 2021	Sweden	Sedentary office workers	43 (8)	34			57	6 months	Reduce, interrupt	Education, environmental, organizations	None	$\dot{V}O_2$max (submaximal, predicted)
McNeil, 2019	Canada	Breast cancer survivors	I1: 58 (9), I2: 58 (10), C 60 (9)	15 (LPA)	15 (HPA)		13	12 weeks	Interrupt	Self-monitoring	lower-intensity PA group: 300 min/wk at 40% to 59% of HR reserve; higher-intensity PA group: 50 min/wk at 60% to 80% of HR reserve	$\dot{V}O_2$max (submaximal, predicted)
Patel, 2022	India	Desk-based office workers	37.3	27			19	4 weeks	Reduce work	Education	30 min walking x 5 /wk	$\dot{V}O_2$max (submaximal, predicted)
Prince, 2018	Canada	Cardiac rehabilitation patients	I: 62.4 (10.7),	17			21	8 weeks	Interrupt	Education, prompts	2 x 1-h/wk (both groups)	$\dot{V}O_2$peak (submaximal, predicted)

			C: 61.5 (9.7)								
Reich, 2020	Austria	Hospital employees	46 (9)	23 (IG-PT)	22 (IG-C)	17	1 year	Reduce work	Environmental	≥ 150 min/wk of MVPA during commute	Watts (maximal)

6MWT – 6 minute walk test, ASP – afterschool program intervention, C – control, CRF – cardiorespiratory fitness, EX – exercise intervention, EX-rST – combined aerobic and exercise training and reduced sedentary behaviour intervention, h – hour, HPA – higher-intensity physical activity intervention, HR –heart rate, I – intervention, LPA – lower-intensity physical activity intervention, METS – metabolic equivalents of tasks, MVPA – moderate-to-vigorous intensity physical activity, PA – physical activity, rST – reduced sedentary behaviour intervention, SPE – school physical education intervention, SPE + ASP – school physical education and afterschool program intervention, UK – United Kingdom, USA – United States of America

Table S5. Quasi-experimental study characteristics

First author, year	Country	Study design	Population description	Age at baseline (SD)	Sample size analyzed				I length, follow-up	SB target/ exposure	Intervention strategy	PA intervention target or exposure adjustment	CRF outcome
					I or Cohort								
**Children and youth <18 years**													
Epstein, 2000	USA	4-arm non-controlled trial	Eight- to twelve-year-old children with obesity	10.5 (1.2)	19	19	19	20	6 months	Reduce total ST	Educational	Increased PA	$\dot{V}O_2$max
Gow, 2016	Australia	Pre-post	Adolescents with obesity and pre-diabetes or insulin resistance	13.1 (1.9)	111				12 months	Reduced screen time	Educational	Phase I and III: aim of spending ≥ 60 min per day in MVPA. Phase II: Two 45-60 min training sessions per week with a qualified personal trainer.	$\dot{V}O_2$peak
Jamerson, 2017	USA	Pre-post	Students enrolled in sixth grade (10-12 years)	Black: 12.8 (0.4), non-Black: 12.8 (0.22)	Black: 1035, non-Black: 2778				~7 months	Reduction of screen time	Educational	Be active every day	Resting & recovery HR
**Adults ≥18 years**													
Aguiñaga, 2021	USA	Pre-post	Older Latinos attending an adult wellness centre	67 (7.1)	20				16 weeks	Interrupt ST	Educational, prompts, self-monitoring	2 h/week of dance	METS
Freene, 2020	Australia	Pre-post	Cardiac rehabilitation patients	54 (13)	19				6 weeks	Reduce total ST	Educational, prompts, self-monitoring	Cardiac rehab	6MWT distance
Overgaard, 2018	Denmark	2-arm non-controlled trial	Inactive and obese participants	Sit-Less: 45.0 (11.5), ExMore: 46.1 (10.3)	23		20		4 weeks	Reduce total ST	Educational, self-monitoring	ExMore group instructed to increase MVPA to ≥ 30 min/day	$\dot{V}O_2$max
Peterman, 2019	USA	Pre-post	Office workers who sat at work >6 h/day	34.8 (9.9)	21				4 weeks	Reduce & interrupt ST	Educational, environmental	Cycling device at the desk, encouraged to use device for ≥ 15 min/h, which for a typical 8-h workday would result in ≥ 2 h of cycling	$\dot{V}O_2$max
Pippi, 2022	Italy	Pre-post	Adults with overweight/obesity	53.08 (9.07)	293				3 months	Reduce total ST	Educational	25 bi-weekly 90-minute session of aerobic and strength training	$\dot{V}O_2$max

6MWT – 6-minute walk test, CRF – cardiorespiratory fitness, h – hour, HR – heart rate, I – intervention, METS – metabolic equivalents of tasks, MVPA – moderate-to-vigorous intensity physical activity, NA – not applicable, PA – physical activity, ST – sedentary time, UK – United Kingdom, USA – United States of America

Table S6. Prospective cohort study characteristics

First author, year	Country	Study design	Population description	Age at baseline (SD)	Sample size analyzed	I length, follow-up	SB target/ exposure	Intervention strategy	PA intervention target or exposure adjustment	CRF outcome
					I or Cohort					
**Children and youth <18 years**										
Aggio, 2012	UK	Cohort	School children	11.5 (0.5)	1500	2 years	Screen time	NA	NA	Laps
Beltran-Valls, 2021	Spain	Cohort	Adolescents	13.9 (0.3)	189	2 years	ST	NA	NA	Laps
Hancox, 2004	New Zealand	Cohort	Children who resided in Otago	5	657	26 years	Screen time	NA	MVPA adjustment	$\dot{V}O_2max$
Haynes, 2022	Australia	Cohort	Participants were Generation 2 (Gen2) of the Raine Study, whose pregnant mothers were recruited between May 1989 and November 1991	5	449	23 years	Screen time	NA	NA	$\dot{V}O_2peak$
Leppanen, 2017	Sweden	Cohort	Preschool children (4 years)	4.48 (0.15)	138	12 months	ST	NA	Vigorous PA adjustment	Laps
Lobelo, 2009	USA	Cohort	School-aged girls from grade 8 to 12	13.6 (0.6)	2101	4 years	Screen time	NA	NA	$\dot{V}O_2max$
Mitchell, 2012	USA	Cohort	A cohort of US students were enrolled in sixth grade and followed to end of eighth grade.	Boys: 11.4 (0.6) Girls: 11.3 (0.6)	2097	2 years	Screen time	NA	Vigorous PA adjustment	Laps
Mota, 2010	Portugal	Cohort	Students enrolled in 2nd grade and followed to 4th grade.	7	135	2 years	TV time	NA	NA	Distance
Potter, 2018	Canada	Cohort	Children aged 4-5 attending a preschool immunization appointment	4.5 (0.5)	649	3 years	Screen time	NA	NA	$\dot{V}O_2max$
Resiberg, 2020	Estonia	Cohort	Children aged 6 to 7 years who were in their last preschool year at baseline, and in transition to grade school	6.6 (0.51)	147	12 months	ST	NA	Vigorous PA adjustment	Laps
Santos, 2018	Portugal	Cohort	Children were from fifth-grade classes	10.55 (0.56)	315	16 months	ST	NA	Light, moderate and vigorous PA adjustment	$\dot{V}O_2max$
**Adults ≥18 years**										
Gomez-Bruton, 2020	Spain	Cohort	Older adults	M: 70.3 (4.3). W: 70.7 (4.7)	642	8 years	Sitting time	NA	NA	6MWT distance
Knaeps, 2018	Belgium	Cohort	Community dwelling Flemish adults (18-75 years)	46.13 (9.54)	524	10 years	ST	NA	NA	$\dot{V}O_2peak$

Nayor, 2021	USA	Cohort	Predominantly middle-aged community-dwelling Framingham Heart Study participants	54 (9)	2070	7.8 years	ST	NA	MVPA adjustment	$\dot{V}O_2$peak
Saidj, 2016	Denmark	Cohort	Community dwelling adults (18-69 years)	44	1403	5 years	Sitting time	NA	MVPA adjustment	$\dot{V}O_2$max

6MWT – 6-minute walk test, CRF – cardiorespiratory fitness, h – hour, HR – heart rate, I – intervention, M – men, METS – metabolic equivalents of tasks, MVPA – moderate-to-vigorous intensity physical activity, NA – not applicable, PA – physical activity, ST – sedentary time, UK – United Kingdom, USA – United States of America, W – women
