# Supplementary material for: The Effect of Sedentary Behaviour on Cardiorespiratory Fitness: A Systematic Review and Meta-Analysis
Source: Sports Med. 2024 Jan 16;54(4):997–1013. doi: 10.1007/s40279-023-01986-y (PMC11052788; doi:10.1007/s40279-023-01986-y)
Supplement: Supplementary file 5 — Supplementary file5 (PDF 223 KB) [file 40279_2023_1986_MOESM5_ESM.pdf]

Title: The effect of sedentary behaviour on cardiorespiratory fitness: a systematic review and meta-analysis.
Journal: Sports Medicine
Authors: Stephanie A. Prince*, Paddy C. Dempsey, Jennifer L. Reed, Lukas Rubin, Travis J. Saunders, Josephine Ta, Grant R. Tomkinson, Katherine Merucci, Justin J. Lang
***Corresponding author:** Centre for Surveillance and Applied Research, Public Health Agency of Canada, stephanie.prince.ware@phac-aspc.gc.ca

Table S7a. Risk of bias of randomized controlled trials.

Author, year	RoB arising from the randomization process	RoB due to deviations from the intended interventions	RoB due to missing outcome data	RoB in measurement of the outcome	RoB in selection of the reported result	Overall RoB judgement
Aguinaga, 2019	Some concerns	Some concerns	Some concerns	High	Some concerns	High
Balducci, 2019	Low	Low	Low	Low	Low	Low
Bergman, 2018	Low	Low	Some concerns	Low	Low	Some concerns
Carr, 2013	Some concerns	Some concerns	High	Some concerns	Low	High
Carr, 2016	Low	Low	Low	Low	Low	Low
Carter, 2020	High	High	High	High	High	High
Cheng, 2022	Low	High	Low	Low	Low	High
Dunning, 2018	Low	Some concerns	Some concerns	Low	Low	Some concerns
Guirado, 2022	Some concerns	Some concerns	Some concerns	Some concerns	Low	Some concerns
Kozey-Keadle, 2014	High	Some concerns	Low	Low	Low	High
Larisch, 2021	Low	Low	Some concerns	Low	Low	Some concerns
McNeil, 2019	Some concerns	Some concerns	Low	Low	Low	Some concerns
Patel, 2022	Low	Low	Low	Some concerns	Low	Low
Peralta, 2009	Low	Low	Some concerns	Low	Low	Low
Prince, 2018	Low	Some concerns	Low	Low	Low	Low
Reich, 2020	Some concerns	Some concerns	Low	High	Some concerns	High
Robinson, 1999	Low	Some concerns	Low	Low	Low	Some concerns
Sacher, 2010	Low	Some concerns	High	High	Low	High
Zhou, 2019	Low	Some concerns	Some concerns	Low	Low	Some concerns

RoB – risk of bias

Table S7b. Risk of bias of quasi-experimental studies.

Author, year	RoB due to confounding	RoB in selection of participants into the study	RoB in classification of interventions	RoB due to deviation from intended interventions	RoB due to missing data	RoB arising from measurement of outcomes	RoB in selection of the reported result	Overall RoB judgement
Aguiñaga, 2021	Serious	Moderate	Moderate	Serious	Low	Critical	Serious	Critical
Eppstein, 2000	Low	Moderate	Serious	Low	Low	Critical	Low	Critical
Freene, 2020	Critical	Moderate	Moderate	Serious	Serious	Moderate	Low	Critical
Gow, 2016	Moderate	Low	Low	Moderate	Moderate	Low	Serious	Serious
Jamerson, 2017	Critical	Low	Low	Critical	Critical	Critical	Moderate	Critical
Overgaard, 2018	Critical	Moderate	Moderate	Low	Serious	Low	Low	Critical
Peterman, 2019	Critical	Critical	Moderate	Moderate	Low	Serious	Low	Critical
Pippi, 2022	Serious	Low	Low	Low	Low	Moderate	Low	Serious

RoB – risk of bias

Table S7c. Risk of bias of cohort studies.

**Author, year**	**RoB due to confounding**	**RoB arising from measurement of the exposure**	**RoB in selection of participants into the study (or into the analysis)**	**RoB due to post-exposure interventions**	**RoB due to missing data**	**RoB arising from measurement of outcomes**	**RoB in selection of the reported result**	**Overall RoB judgement**
Aggio, 2023	Very high	Very high	Very high	Some concerns	High	Low	High	Very high
Beltran-Valls, 2021	High	Some concerns	Some concerns	High	High	Some concerns	Some concerns	High
Gomez-Bruton, 2020	Some concerns	Some concerns	Low	High	High	Low	Low	High
Hancox, 2004	Low	Some concerns	Low	Some concerns	Some concerns	Low	Low	Some concerns
Haynes, 2022	Low	Some concerns	Low	Some concerns	Some concerns	Low	Low	Some concerns
Knaeps, 2018	Very high	Some concerns	High	High	Some concerns	Low	Some concerns	High
Leppanen, 2017	Low	Low	Low	Some concerns	Low	Low	Some concerns	Some concerns
Lobelo, 2009	High	High	High	Very high	Very high	Some concerns	Some concerns	High
Mitchell, 2023	Low	Some concerns	Low	Low	Low	Some concerns	Some concerns	Some concerns
Mota, 2010	High	Some concerns	Low	Low	Low	Some concerns	Low	Some concerns
Nayor, 2021	Low	Low	Low	Low	Very high	Low	Some concerns	Some concerns
Potter, 2018	High	Some concerns	Low	Low	Very high	Low	Some concerns	Very high
Resiberg, 2020	Low	Low	Low	Low	High	Low	Some concerns	High
Saidj, 2016	Low	Low	Some concerns	Low	Low	Low	Low	Some concerns
Santos, 2018	Low	Lo	Some concerns	Some concerns	Some concerns	Low	Low	Some concerns

RoB – risk of bias
