# Supplementary material for: The Effect of Sedentary Behaviour on Cardiorespiratory Fitness: A Systematic Review and Meta-Analysis
Source: Sports Med. 2024 Jan 16;54(4):997–1013. doi: 10.1007/s40279-023-01986-y (PMC11052788; doi:10.1007/s40279-023-01986-y)
Supplement: Supplementary file 6 — Supplementary file6 (PDF 331 KB) [file 40279_2023_1986_MOESM6_ESM.pdf]

Title: The effect of sedentary behaviour on cardiorespiratory fitness: a systematic review and meta-analysis.
Journal: Sports Medicine
Authors: Stephanie A. Prince*, Paddy C. Dempsey, Jennifer L. Reed, Lukas Rubin, Travis J. Saunders, Josephine Ta, Grant R. Tomkinson, Katherine Merucci, Justin J. Lang
***Corresponding author:** Centre for Surveillance and Applied Research, Public Health Agency of Canada, stephanie.prince.ware@phac-aspc.gc.ca

Table S8. Randomized controlled trial intervention effects on SB, PA and CRF among youth.

First author, year	Change in SB	Change in PA (MVPA where possible)	Change in CRF	Overall RoB
**SB-only targeted interventions**				
Robinson, 1999	Significant decrease in TV/video games in intervention vs. control, $p < 0.001$	NS b/w group difference in MVPA	NS b/w group differences, $p = 0.45$	Some concerns
**PA targeted interventions**				
Peralta, 2009	NS b/w group differences in leisure screen change	NS b/w group differences in change of weekday or weekend MVPA	NS b/w group differences in change, $p = 0.61$	Low
Sacher, 2010	Significant ↓ in intervention vs. CON	Significant ↑ in intervention vs. CON	Significant ↑ in intervention vs. CON, $p = 0.003$	High
Zhou, 2019	Significant ↓ in TV and video games during school days in interventions, NS in CON<br>Significant ↑ in %SB in 2 intervention groups, NS in one intervention and CON	Significant ↑ in all intervention groups, NS in CON	Significant ↑ in all interventions vs. CON, $p < 0.0001$	Some concerns

bpm – beats per minute, b/w – between, CON – control group, HR – heart rate, NS – not statistically significant $p<0.05$, MVPA – moderate-to-vigorous intensity physical activity, PA – physical activity, SB – sedentary behaviour

Table S9. Randomized controlled trial intervention effects on SB, PA and CRF among adults.

First author, year	Change in SB	Change in PA (MVPA where possible)	Change in CRF	Overall RoB
**SB-only targeted interventions**				
Carr, 2013	Min/day: significant ↓ in intervention, significant ↑ in control<br>b/w-group difference favours intervention, p = 0.01<br><br>%/day: significant ↓ in intervention, NS for control<br>NS b/w-group difference, p = 0.06	Min/day MPA: No change in intervention or control<br>NS b/w-group difference, p = 0.13<br><br>%/day MVPA: significant ↑ in intervention, NS for control<br>NS b/w-group difference, p=0.06	NS difference over time in each group<br>NS b/w-group difference, p = 0.10	High
Carr, 2016	NS change in either group for occupational sitting.<br>Cycle erg: -2.0, p = 0.09<br>Erg workstation: +0.4, p = 0.57	NS change in either group for occupational PA.<br>Cycle erg: MPA %:1.1, p = 0.32, VPA % : 0.3, p = 0.10<br>Erg workstation: MPA%: 0.07, p = 0.85, VPA%: 0.0, p = 0.84	NS intervention effect on resting HR, p = 0.32	Low
Cheng, 2022	NS change in either group (both decreased) for min/day or % day<br>NS b/w-group difference, p = 0.81/0.84	Sig ↑ in intervention (+8 min/day), NS control<br>NS b/w group difference, p = 0.07	NS change in intervention, sig increase in control (+18 m)<br>NS b/w -group difference, p=0.41	High
Dunning, 2018	NS group by time interaction, p = 0.76	NS group by time interaction, p = 0.11	Between group differences not described, but data suggests no difference in post-values.	Some concerns
Kozey-Keadle, 2014	NS diff in Ex group, Sig. ↓ in Ex-rST and rST, sig ↑ in control<br>b/w-group differences:<br>EX-rST<EX; rST, EX-rST, EX < CON	Sig ↑ in Ex, Ex-rST and rST, NS in control<br>b/w-group differences:<br>EX-rST>rST, EX, CON; rST, EX > CON	Sig ↑ in EX and EX-rST groups, NS in rST and CON	High
Larisch, 2021	NS group x time interaction	NS group x time interaction	Sig. ↑ in PA group, NS change in SED or CON groups<br>NS b/w differences	Some concerns
Prince, 2018	NS b/w group differences in change	NS b/w group differences in change	NS b/w group differences in change	Low
**PA targeted interventions**				
Aguinaga, 2019	NS change over time p = 0.26<br>NS time by group p = 0.47	NS change over time p = 0.27<br>NS time by group p = 0.57	↑ in both groups over time p = 0.001<br>Time by group p = 0.49	High
Balducci, 2019	Significant ↓ in both groups<br>Mean b/w group difference favours intervention -0.7 h/day, p < 0.001	Significant ↑ in both groups<br>Mean b/w group difference favours intervention 6.8 min/day, p < 0.001	Significant ↑ in both groups<br>Mean b/w group difference favours intervention 1.4 ml/min/kg, p = 0.001	Low
Bergman, 2018	NS difference in sitting at 13 months for either group (sig in I group at 2, 6 & 10 months)<br>NS group x time interaction	Significant ↓ in MVPA both groups<br>NS group x time interaction	NS difference in RHR in either group.<br>NS group x time interaction.	Some concerns
Carter, 2020	↑ in control, ↓ in intervention<br>b/w group difference is large, -38.2 min/8-h workday	Minimal change in both groups<br>Small difference b/w groups	No change in control, small reduction of 1 bpm in intervention<br>Small b/w difference (NS)	High

Guirado, 2022	Min/day: NS change in either group<br>NS group x time interaction<br>%/day: Significant ↓ in control, NS in intervention<br>NS group x time interaction	NS change in either group<br>NS time by group interaction	Significant ↑ in intervention, NS in control<br>NS group by time interaction, p = 0.375	Some concerns
McNeil, 2019	Low-intensity PA group: sig. ↓ p = 0.01<br>High intensity PA group: NS change<br>Control: NS change<br>Significant group x time for low intensity vs. control	Low-intensity PA group: sig. ↑ p < 0.001<br>High intensity PA group: sig ↑ p = 0.01<br>Control: NS change<br>Significant group x time for low intensity vs. control	Low-intensity PA group: Significant ↑ p < 0.01<br>High intensity PA group: Significant ↑ p < 0.01<br>Control: NS change<br>Significant group x time for low & high intensity vs. control	Some concerns
Patel, 2022	NS difference in intervention or CON<br>NS b/w group differences	NS difference in intervention or CON<br>NS b/w group differences	NS difference in intervention or CON<br>NS b/w group differences	Low
Reich, 2020	Significant ↓ in cycling group, NS in public transit or CON	Significant ↑ in both intervention groups, NS in CON	Significant ↑ in both intervention groups, decrease in CON	High

bpm – beats per minute, b/w – between, CON – control group, EX – exercise intervention, EX-rST – combined aerobic and exercise training and reduced sedentary behaviour intervention, HR – heart rate, MPA – moderate-intensity physical activity, MVPA – moderate-to-vigorous intensity physical activity, NS – not statistically significant p<0.05, PA – physical activity, RHR – resting heart rate, SED – reduced sedentary behaviour intervention, VPA – vigorous-intensity physical activity

Table S10. Quasi-experimental study effects on SB, PA and CRF among youth.

First author, year	Change in SB	Change in PA (MVPA where possible)	Change in CRF	Association between SB and CRF	Association between ↓ SB and CRF	Overall RoB
Epstein, 2000	Targeted low dose increased PA arm. Targeted SB (%): -6.5 (17.3). Non-targeted SB (%): 7.4 (17.6)<br><br>Targeted high dose increased PA arm. Targeted SB (%): -9.4 (18.0). Non-targeted SB (%): 8.2 (18.0)<br><br>Targeted low dose reduced SB arm. Targeted SB (%): -15.1 (19.0). Non-targeted SB (%): 11.1 (24.7)<br><br>Targeted high dose reduced SB arm. Targeted SB (%): -20.3 (29.4). Non-targeted SB (%): 10.5 (17.8)	Targeted low dose increased PA arm. PA time (%): -0.9 (10.8).<br><br>Targeted high dose increased PA arm. PA time (%): 1.2 (12.7)<br><br>Targeted low dose reduced SB arm. PA time (%): 4.0 (11.0)<br><br>Targeted high dose reduced SB arm. PA time (%): 9.7 (17.7)	Targeted low dose increased PA arm. PWC150 (kpm/min): 0-6 months: 135.3 (95.5) FFM-adjusted PWC150 (kpm/min/FFM): 3.6 (2.1)<br><br>Targeted high dose increased PA arm. PWC150 (kpm/min): 110.9 (88.6); FFM-adjusted PWC150 (kpm/min/FFM): 3.3 (2.4)<br><br>Targeted low dose reduced SB arm. PWC150 (kpm/min): 113.3 (82.1); FFM-adjusted PWC150 (kpm/min/FFM): 3.0 (2.1)<br><br>Targeted high dose reduced SB arm. PWC150 (kpm/min): 95.2 (90.8); FFM-adjusted PWC150 (kpm/min/FFM): 2.5 (2.3)	Targeted SB showed a significant decrease from baseline at 6 months ($p < 0.001$). Nontargeted SBs were increased from baseline at 6 months ($p < 0.05$). The results provide experimental evidence that reducing access to SB is an alternative to targeting PA. The 2 approaches were associated with similar increases in fitness during the observation.	↓ SB = ↑ CRF	Critical
Gow, 2016	Leisure screen time:<br>-49 min/day, 95% CI: -23 to -74	MVPA: +19 min/day, 95% CI: 5 to 23, p = 0.028	+5.9%, p = 0.018	$\dot{V}O_2$peak and time to fatigue improved at 6 months. Changes in MVPA correlated with CRF, but no significant association between changes in SB and CRF.	SB ≠ CRF	Serious
Jamerson, 2017	TV time:<br>Black: -0.18, p = 0.01, non-Black: -0.17, p < 0.0001<br>Computer time:<br>Black: 0.07, p = 0.2, non-Black: -0.02, p = 0.7<br>Video game time:	Moderate PA<br>Black: 0.2, p = 0.03<br>non-Black: 0.26, p < 0.0001<br><br>Vigorous PA<br>Black: 0.31, p = 0.0008<br>non-Black: 0.31, p < 0.0001	Resting HR:<br>Black: -0.80, 95% CI: -1.88 to 0.28, p = 0.1, non-Black: -0.77, 95% CI: -1.38 to -0.15, p = 0.02<br><br>Recovery HR:	Both Black and non-Black students saw improvements in their leisure screen time and PA levels. Resting HR only significantly reduced in non-Black students, while recovery HR significantly improved in Black students only.	SB ? CRF (unclear as PA increased)	Critical

	Black: -0.04, p = 0.6, non-Black: -0.1, p < 0.0001		Black: -3.6, 95% CI: -5.3 to -1.9, p < 0.0001, non-Black: =0.51, 95% CI: -1.47 to 0.45, p = 0.3			

CI – confidence interval, CRF – cardiorespiratory fitness, HR – heart rate, MVPA – moderate-to-vigorous intensity physical activity, PA – physical activity, SB – sedentary behaviour

Table S11. Quasi-experimental study effects on SB, PA and CRF among adults.

First author, year	Change in SB	Change in PA (MVPA where possible)	Change in CRF	Association between SB and CRF	Association between ↓ SB and CRF	Overall RoB
Aguiñaga, 2021	Device ST: +24.6 min/day, 95% CI: -16.1 to 65.3, p = 0.235<br><br>Self-rep weekday: +0.3 min/week, 95% CI: -0.4 to 1.0, p = 0.605<br><br>Self-rep weekend: +0.3 min/week, 95% CI: -0.5 to 1.1, p = 0.579	Device MVPA: +34.7 min/day, 95% CI: 17.7 to 51.7, p = 0.015<br><br>Self rep: +208.9 min/week, 95% CI: 141.2 to 276.6, p = 0.002	METS: +2.1, 95% CI: 1.4 to 2.8, p = 0.001	Participants significantly improved their PA levels and CRF, but no significant change in SB.	SB ≠ CRF	Critical
Freene, 2020	Pre: SB minutes: 747 (224), % SB: 68.2 (9.9), bout duration: 23 (5.7), # bouts/day: 16 (6.5), # SB breaks: 15 (6.5)<br><br>6-weeks: SB min/day: 774 (209), % SB: 68.8 (9), bout duration: 24 (4.8), #bouts/day: 17 (7.1), # SB breaks: 16 (7.1)	Pre: 74 (23) min/day, 6 weeks: 78 (27) min/day	Pre: 506 (83) m, 6-weeks: 581 (75) m, p < 0.001	SB appears to have decreased, and CRF improved over 6-weeks. Not clear if PA significantly changed.	SB ? CRF (unclear if PA changed)	Critical
Overgaard, 2018	Sit less: -53 min/day, 95%CI: -10 to -96	Exercise more: +16 min/day, 95% CI: 5 to 27	Sit less: +1.9, 95% CI: 0.3 to 3.5, +8%<br><br>Move more: +2.2, 95% CI: 0.8 to 3.7, +11%	Completion of two 4-week advice-based intervention induced changes in PA in obese men and women. The reported changes were specific to the advice given in the two intervention groups, such that the Exercise More group spent more time in MVPA, whereas the Sit Less group reduced ST.	↓ SB = ↑ CRF	High
Peterman, 2019	Pre = 583.2 (59.5), post = 566.2 (85.7) min/day.	Self-reported daily cycling averaged 1.77 (0.48) h/day with a range of 0.99 to 2.82 h/day. Noncycling PA remained constant during both workdays and non-	+0.10, 95% CI: -0.17 to 0.37, p<0.01	A stationary cycling device incorporated into a sedentary workplace for 4 weeks improves fitness.	SB ? CRF (unclear if it's the added PA from cycling as SB changed by 20 min/day)	Critical

		workdays. MVPA pre = 34.5 (17.1), post = 37.8 (19.9)				
Pippi, 2022	Sitting time pre: 5.36 (3.17) h/day, post: 5.15 (2.69) h/day, t = -1.056, p = 0.146	MVPA pre: 16.46 (24.71) MET-h/week, post: 39.80 (27.88) MET-h/week, t = 12.372, p<0.001	$\dot{V}O_2$max pre: 19.52 (9.30), post: 25.93 (7.93), t = 16.409, p<0.001	No significant time x group interaction for sitting or MET-h/week and CRF.	SB ≠ CRF	Serious

CI – confidence interval, CRF – cardiorespiratory fitness, MVPA – moderate-to-vigorous intensity physical activity, PA – physical activity, SB – sedentary behaviour, ST – sedentary time

Table S12. Cohort study associations between SB and CRF among youth

First author, year	Change in SB	Change in PA (MVPA where possible)	Change in CRF	Association between SB and CRF	Association between ↓ SB and CRF	Overall RoB
Aggio, 2012	NA	NA	NA	High vs. low baseline screen time was associated with greater likelihood of becoming unfit (OR = 2.35, 95% CI: 1.40 – 4.00)	↓ SB = ↑ CRF	Very high
Beltran-Valls, 2021	NA	NA	NA	Low sedentary time, compared to high, was significantly associated with high CRF at follow-up (aOR* = 3.09, 95%CI: 1.13 to 8.48) *Note PA not adjusted for in analysis.	↓ SB = ↑ CRF	High
Hancox, 2004	NA	NA	NA	Childhood and adolescent (age 5 to15 years) TV viewing predicted lower $\dot{V}O_2$max at age 26 years (β = -0.12, SE = 0.04, p = 0.0009). Similar patterns of results were seen for childhood (age 5 to 11 years), adolescence (age 13 to 15 years), and early adulthood (age 21 years). Additional adjustment for reported PA at age 15 years did not change the association between child and adolescent TV viewing and $\dot{V}O_2$max at age 26 years.	↓ SB = ↑ CRF*	Some concerns
Haynes, 2022	NA	NA	NA	TV watching throughout childhood and adolescence predicts CRF in adulthood. The impact of TV watching during childhood and adolescence on adult CRF is largely negated by current PA levels in adulthood. The negative impact of high TV watching in childhood and adolescence was not present in those engaging in higher PA levels as an adult, whilst the legacy impact of low TV viewing does not protect individuals from the detrimental impact of low current PA levels.<br><br>There was a step-wise increase in CRF (time to exhaustion) from High TV to Low TV trajectories:<br>• High TV vs Increasing TV +0.8 (0.3) min^{-1}, p = 0.020<br>• Increasing TV vs Low TV +0.9 (0.4) min^{-1}, p = 0.042<br>• High TV vs Low TV +1.6 (0.4) min^{-1}, p<0.001.<br><br>The trend for step-wise increases in CRF between trajectories were similar for $\dot{V}O_2$peak (mL/kg/min), although not all between-group differences were statistically significant:<br>• High TV vs Increasing TV +1.8 (0.9) mL/kg/min, p = 0.057<br>• Increasing TV vs Low TV +2.6 (1.2) mL/kg/min, p = .038<br>• High TV vs Low TV +4.4 (1.2) mL/kg/min, p<0.001	SB ≠ CRF	Some concerns

				There were no TV trajectory*sex interaction effects for TTE (p = 0.146) or V̇O_2peak mL/kg/min [1] (p = 0.373). Linear regression indicated there were no significant interaction effects for TV trajectory*IPAQ category (F = 2.76, p = 0.064), indicating the way CRF was impacted by current PA was not different based on TV trajectory membership.		
Leppanen, 2017	NA	NA	NA	No significant association between baseline SB and 12-month CRF. Adjusted β = 0.01, 95% CI: -0.10 to 0.10, p = 0.93	SB ≠ CRF*	Some concerns
Lobelo, 2009	NA	NA	NA	Elevated exposure to screen time (≥2 hours/day) was associated, in a cross-sectional and longitudinal fashion, with lower CRF in adolescent girls.	↓ SB = ↑ CRF	High
Mitchell 2012	Screen time<br>Boys baseline: 3.55 (2.50) h/day, follow-up: 4.00 (2.38) h/day<br><br>Girls baseline: 3.44 (2.43) h/day, follow-up: 3.67 (2.40) h/day	Vigorous PA<br>Boys baseline: 95.3 (79.3) min/day, follow-up: 73.5 (72.5) min/day<br><br>Girls baseline: 83.9 (72.2) min/day, follow-up: 57.2 (64.7) min/day	CRF<br>Boys baseline: 23.7 (13.9) laps, follow-up: 35.0 (19.4) laps<br><br>Girls baseline: 19.3 (10.2) laps, follow-up: 20.9 (11.6) laps	More screen time was associated with lower CRF from age 11 to 13, independent of vigorous PA. However, the association was weakest at the lower tail of the CRF distribution.<br><br>In boys, more screen time was associated with fewer shuttle run laps completed from age 11 to 13 at the 25th, 50th, and 75th shuttle run lap percentiles; the strongest association was at the 75th shuttle run percentile (β = -0.57, 95% CI: -0.93 to -0.21).<br><br>In girls, more screen time was associated with fewer shuttle run laps completed from age 11 to 13 at the 50th, 75th, and 90th shuttle run lap percentiles; the strongest association was at the 90th shuttle run percentile (β = -0.65, 95% CI: -1.01 to -0.30).<br><br>Borderline negative associations were found between screen time and shuttle run laps at the 10th shuttle run percentile in boys and girls (β = -0.28, 95% CI: -0.57 to 0.01 and β = -0.17, 95% CI: -0.41 to 0.06, respectively).	↓ SB = ↑ CRF*	Some concerns
Mota, 2010	NA	NA	NA	Baseline TV watching (low vs. high TV) and change in CRF OR = 0.36, 95% CI: 0.15 to 0.86, p = 0.02. High TV vs. low TV and follow-up CRF OR = 2.48, 95% CI: 1.05 to 5.89, p = 0.04<br><br>The findings of this study suggest that there was a significant inverse association between times spent watching TV and CRF over a 2-yr period.	↓ SB = ↑ CRF	Some concerns
Potter, 2018	NA	NA	NA	No significant associations were observed between baseline ST and follow-up CRF (β = -0.003, 95% CI: -0.018 to 0.012, p = 0.669). Similarly, changes in ST were not significantly associated with CRF at follow-up (β = 0.003, 95% CI: -0.012 to 0.018, p = 0.731).	SB ≠ CRF	Very high

Reisberg, 2020	Device ST baseline: 414 (96) min/day, follow-up: 459 (91.6) min/day, p <0.001	Device MVPA baseline: 68.6 (25) min/day, follow-up: 74.2 (25.9) min/day, p = 0.006	Baseline: 19.8 (9.57) laps, follow-up: 23.7 (13.53), p<0.001	No significant associations between SB and the number of laps in the 20-m shuttle run test were detected in unadjusted models (*b* = -0.005, 95% CI: -0.034 to 0.024, p = 0.722), but in adjusted models, SB positively associated (*b* = 0.006, 95% CI: -0.062 to 0.075, p = 0.034) with the number of laps.	↑ SB = ↑ CRF*	Some concerns
Santos, 2018	Device ST baseline: 526.99 (66.80) min/day, follow-up: 543.20 (74.37) min/day, p<0.001	Device MVPA baseline: 60.45 (24.45) min/day, follow-up: 53.62 (24.12) min/day, p<0.001	$\dot{V}O_2$max baseline: 35.5 (7.4) mL/kg/min, follow-up: 36.6 (6.8) ml/kg/min, p<0.001	Unadjusted and adjusted associations were not significant between ST and CRF (β = 0.001, 95% CI: 0.000 to 0.001). Replacing SB with LPA or MPA was not prospectively associated with change in CRF. Replacing SB with VPA was associated with prospective increase in CRF.	SB ≠ CRF*	Some concerns

* estimates adjusted for physical activity, aOR – adjusted odds ratio, CI – confidence interval, CRF – cardiorespiratory fitness, LPA – light-intensity physical activity, MPA – moderate-intensity physical activity, MVPA – moderate-to-vigorous intensity physical activity, NA – not available, PA – physical activity, SB – sedentary behaviour, ST – sedentary time, VPA – vigorous intensity physical activity

Table S13. Cohort study associations between SB and CRF among adults

First author, year	Change in SB	Change in PA (MVPA where possible)	Change in CRF	Association between SB and CRF	Association between ↓ SB and CRF	Overall RoB
Gomez-Bruton, 2020	Hours sitting per day were used to classify subjects into non-sedentary (< 4 h/day) vs. sedentary (≥ 4 h/day)	NA	NA	There were no differences in the reductions in fitness for the sedentary and non-sedentary groups for men. Sedentary women had lower aerobic fitness at both time points, but there was no significant interaction.	SB ≠ CRF	High
Knaeps, 2018	Leisure screen time +2.00 (7.06) h/week, p<0.05<br>Passive transport -0.06 (3.99) h/week, NS	MVPA: -0.59 (4.58) h/week, NS	-1.47 (5.87) mL/kg/min, p<0.05	Changes in ST (r = -0.13, p<0.05) and MVPA (r = 0.20, p <0.05) are independently correlated with positive changes in CRF.	↓ SB = ↑ CRF	High
Nayor, 2021	NA	NA	NA	Reductions in ST resulted in favourable measures of CRF. Reductions of 249 min/day of ST corresponded to a 5% higher $\dot{V}O_2$peak.<br><br>Change in ST and log($\dot{V}O_2$peak) β = -0.054, SE = 0.019, p = 0.008, change in ST and % predicted $\dot{V}O_2$peak β = -0.066, SE = 0.026, p = 0.023	↓ SB = ↑ CRF*	Some concerns
Saidj, 2016	Leisure sitting time pre: 2.9 (1.3) h/day, post: 3.1 (1.3) h/day, p = 0.001<br><br>Occupational sitting time pre: 4.4 (2.6) h/day, post: 4.6 (2.6) h/day, p = 0.0002	MVPA pre: 4.3 (3.8) h/week, post: 4.5 (3.9) h/week, p = 0.0018	$\dot{V}O_2$max pre: 34.6 (8.7) mL/kg/min, post: 35.4 (9.1) mL/kg/min, p = 0.4517	Baseline work sitting predicted a five-year increase in estimated $\dot{V}O_2$max (β = 0.21, 95% CI: 0.08 to 0.32, p<0.01). Higher leisure time sitting at baseline predicted a five-year decrease in estimated $\dot{V}O_2$max (β = -0.26, 95% CI: -0.52 to -0.01, p <0.05).	↓ leisure SB = ↑ CRF*<br><br>↑ work SB = ↑ CRF*	Some concerns

* estimates adjusted for physical activity, CI – confidence interval, CRF – cardiorespiratory fitness, MVPA – moderate-to-vigorous intensity physical activity, NA – not available, PA – physical activity, ST – sedentary time
